# Interferon-stimulated TRIM69 interrupts dengue virus replication by ubiquitinating viral nonstructural protein 3

**DOI:** 10.1371/journal.ppat.1007287

**Published:** 2018-08-24

**Authors:** Kezhen Wang, Chunling Zou, Xiujuan Wang, Chenxiao Huang, Tingting Feng, Wen Pan, Qihan Wu, Penghua Wang, Jianfeng Dai

**Affiliations:** 1 Institutes of Biology and Medical Sciences, Jiangsu Key Laboratory of Infection and Immunity, Soochow University, Suzhou, P. R. China; 2 Key Laboratory of Reproduction Regulation of NPFPC, SIPPR, IRD, Fudan University, Shanghai Institute of Planned Parenthood Research, Shanghai, P. R. China; 3 Department Immunology, School of Medicine, the University of Connecticut Health Center, Farmington, Connecticut, United States of America; National Institute of Allergy and Infectious Diseases, UNITED STATES

## Abstract

In order to eliminate viral infections, hundreds of interferon-stimulated genes (ISGs) are induced *via* type I interferons (IFNs). However, the functions and mechanisms of most ISGs are largely unclear. A tripartite motif (TRIM) protein encoding gene *TRIM69* is induced by dengue virus (DENV) infection as an ISG. TRIM69 restricts DENV replication, and its RING domain, which has the E3 ubiquitin ligase activity, is critical for its antiviral activity. An *in vivo* study further confirmed that TRIM69 contributes to the control of DENV infection in immunocompetent mice. Unlike many other TRIM family members, TRIM69 is not involved in modulation of IFN signaling. Instead, TRIM69 interacts with DENV Nonstructural Protein 3 (NS3) directly and mediates its polyubiquitination and degradation. Finally, Lys104 of NS3 is identified as the target of TRIM69-mediated ubiquitination. Our study demonstrates that TRIM69 restricts DENV replication by specifically ubiquitinating a viral nonstructural protein.

## Author summary

Mosquito-borne viruses, such as Dengue virus (DENV), have become global threats to human health in recent years. However, no antiviral drugs have been approved to treat DENV induced diseases, and the safe and effective vaccines are still under development. It is of great importance to explore the detail mechanisms of host-virus interaction. In this report, we found that an interferon inducible host protein, TRIM69, is upregulated upon DENV infection. TRIM69 acts as a restriction factor for DENV replication both *in vitro* and *in vivo*. As an E3 ubiquitin ligase, TRIM69 directly binds to viral Nonstructural Protein 3 (NS3), which leads to NS3 ubiquitination and degradation. Thus TRIM69 is a novel interferon inducible host antiviral factor by targeting a specific viral protein for its degradation.

## Introduction

Recently, mosquito-borne viral diseases become global threats to human health. As the most significant mosquito-borne viral pathogen, Dengue virus (DENV) is responsible for outbreaks of dengue fever (DF), dengue shock syndrome (DSS), and dengue hemorrhagic fever (DHF). DENV causes millions of infections in over 100 countries annually, resulting in more than 25,000 deaths [1,2]. A DENV vaccine was recently licensed for use after several decades of efforts, however, it confers only partial cross protection for all DENV serotypes [3,4]. Additionally, there is still no antiviral drugs have been approved to treat DENV induced diseases [5,6].

Similar with other mosquito borne flaviviruses, DENV genome is quickly translated into a polyprotein after entering into the host cells. Then the viral polyprotein is cleaved into three structural proteins (capsid protein C, membrane protein M, and envelope protein E) and seven nonstructural proteins (NS1, NS2A, NS2B, NS3, NS4A, NS4B and NS5) [7–10]. The nonstructural proteins are not the component of the mature, infectious virions, but involved in polyprotein processing, viral RNA synthesis, and virus morphogenesis [11]. NS3, a multifunctional protein, has a superfamily 2 (SF2) DEAH-box helicase domain that possesses RNA 5’-triphosphatase (RTPase), RNA-stimulated nucleoside triphosphatase (NTPase), RNA annealing, and 3’-tailed dsRNA unwinding activities [12–17]. It forms a protease complex together with NS2B, helps to cleave the DENV polyprotein and many other proteins, such as STING [18–20]. NS5, containing an N-terminal methyltransferase and a C-terminal RNA-dependent RNA polymerase, is indispensable for viral RNA replication [21,22]. NS4B was recently reported to promote DENV replication and alleviate RIG-I dependent activation of interferon responses by induction of mitochondria elongation [23]. The interaction of NS3 and NS5 is important for DENV RNA replication [24,25]. NS4B also interacts with NS3, facilitating dissociation of NS3 helicase from ssRNA [26–28]. Due to its central role in DENV replication, NS3 is an intriguing target for anti-DENV therapy. Although the functions of other nonstructural proteins remain unclear, they do indeed play important roles in viral replication, assembly and maturation [29].

The tripartite motif family members (TRIMs) share three conserved domains, an N-terminal Really Interesting New Gene (RING) domain, one or two B-Boxes (B1/B2) and a coiled-coil (CC) domain. TRIM proteins are implicated in multiple cellular functions, ranging from transcriptional regulation to post-translational modifications involved in various cellular processes, such as cell differentiation, apoptosis and oncogenesis [30]. TRIM proteins have been long predicted to be part of the innate immune pathway. In line with this, recent studies show that an increasing number of TRIM proteins are recognized as ISGs and mediate antiviral activities [31–33]. The antiviral activities of TRIM proteins depend, for the most part, on their function of E3-ubiquitin ligases activity. TRIM38 sumoylates cGAS and STING during the early phase of virus infection to promote the stability of these two proteins [34]. TRIM56 inhibits bovine viral diarrhea virus (BVDV) replication by targeting intracellular viral RNA replication [35]. TRIM5a is responsible for post-entry restriction of diverse retroviruses, including N-MLV and HIV-1 [32,36]. TRIM5α blocks HIV-1replication by targeting the capsid and promoting its rapid, premature disassembly [37]. At the same time, TRIM5α stimulates innate immune signaling by catalyzing the synthesis of unanchored K63-linked poly-ubiquitin chains that bind and activate TAK1-dependent NF-κB [32]. TRIM22 inhibits HIV-1 by down-regulating the viral long terminal repeat-directed transcription [38,39]. Some TRIM proteins restrict viral replication by directly targeting viral proteins. TRIM22 has been reported to interact with HIV-1 Gag protein, EMCV 3C protease, influenza virus nucleoprotein and HCV NS5A, resulting in inhibition of viral replication [40–43]. TRIM79α inhibits tick-borne encephalitis virus *via* targeting the viral RNA-dependent RNA polymerase, NS5, for lysosomal degradation [44]. Although knowledge on the cellular roles of TRIM E3 ubiquitin ligases has rapidly grown over the last years, many aspects of their molecular functions remain unclear.

Here, we identified another TRIM family member, TRIM69 (also known as RNF36, HSD34, and Trif) as an IFN-inducible virus restriction factor. As an E3 ubiquitin ligase [45], TRIM69 plays crucial roles in apoptosis [46], tumor control [47] and zebrafish development [48,49]. However, TRIM69 has not been reported to have any function on antiviral immunity. In this study, we demonstrated that TRIM69 is an IFN-stimulated gene and restricts DENV replication *in vitro* and *in vivo*. TRIM69 directly interacts with viral NS3 and results in NS3 degradation by proteasomes. Thus, TRIM69 is a novel IFN inducible restriction factor for DENV.

## Results

### TRIM69 is upregulated upon DENV infection

**Fig 1:**
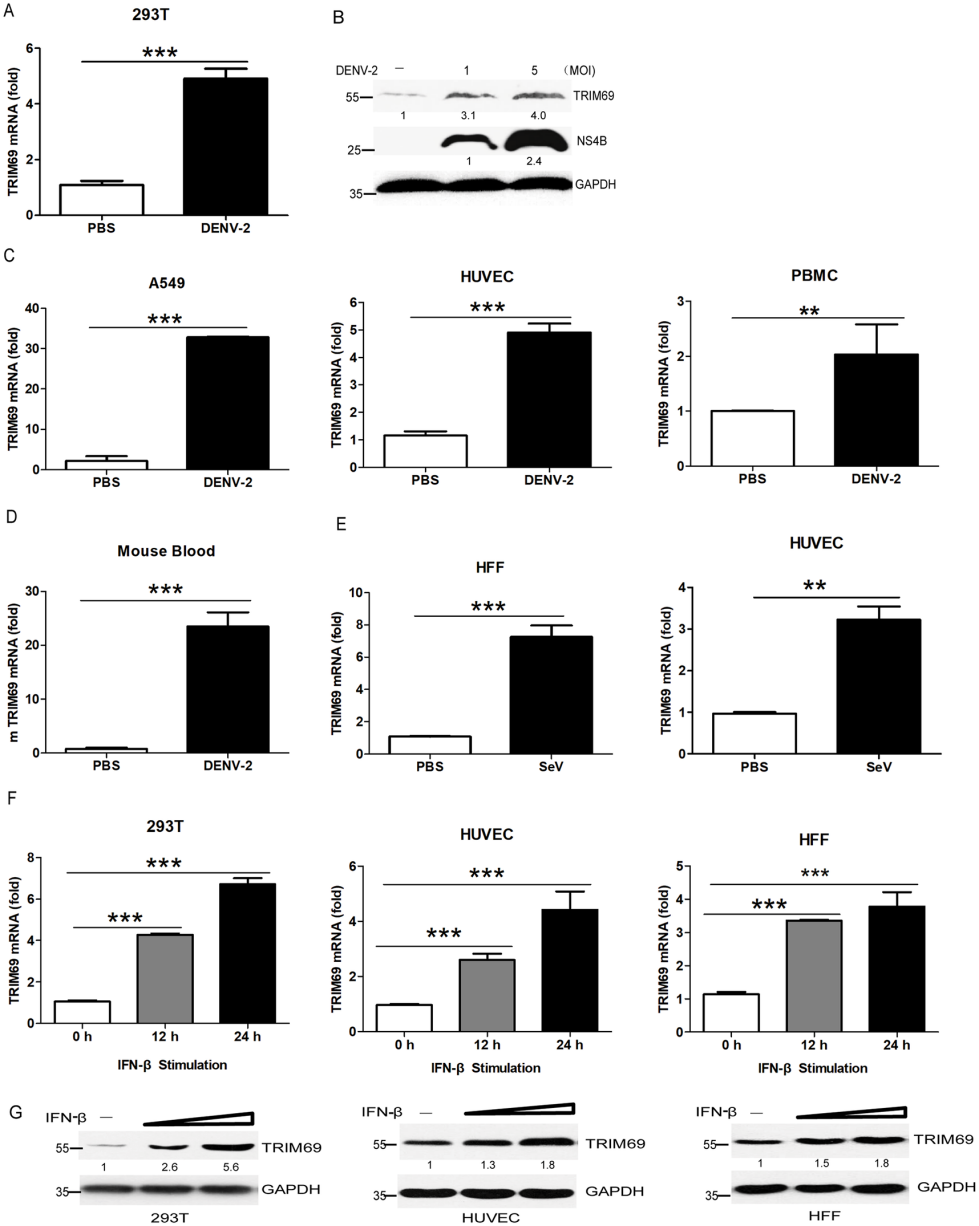
TRIM69 is induced by virus infection or IFN-*β* stimulation. (A, B) TRIM69 mRNA (A) and protein (B) expression in 293T cells with DENV-2 infection. All the cells were harvested at 24 hpi. (C) qRT-PCR analysis of TRIM69 mRNA expression in A549, HUVEC, and PBMC cells infected with DENV-2 at 24 hpi. (D) The expression of TRIM69 in mouse peripheral blood cells infected with DENV-2. Blood samples were collected from mice orbit at 24h post DENV infection. (E) TRIM69 mRNA in HUVEC and HFF cells with SeV infection for 24 h as measured by qRT-PCR. (F, G) RNA (F) and protein expression (G) of TRIM69 in 293T, HUVEC, and HFF cells stimulated by IFN-*β*. All the qPCR results are represented as relative fold changes after normalized to *β*-actin controls. Results are expressed as mean ± SEM. * *p* < 0.05, ** *p* < 0.01, and *** *p* < 0.001. The data shown are representative of at least 3 independent experiments.

To evaluate the mechanisms of how host cells resist a pathogenic microorganism, RNA-Seq was performed to screen out the host factors involved in DENV-2 infection. 152 mRNAs were significantly changed after DENV infection in 293T cells (99 mRNAs were induced, while the others were decreased). As expected, many genes related to antiviral innate immune signal pathway were found out as shown by gene cluster analysis (S1 Fig). Furthermore, 52 of the 99 upregulated genes upon DENV-2 infection were predicted as ISGs by searching the Interferome V2.01 database (www.interferome.org). Many well-known ISGs, such as DDX58, IRF9, ISG15 and STAT1, were screened out after DENV-2 infection (S1 Table). The mRNA expression of *TRIM69*, together with five other putative ISGs (LGALS3BP, C19ORF66, DDX60, FBXO15, and HELZ2) was confirmed to be significantly upregulated after DENV-2 infection (Fig 1A, S2A Fig and S1 Table). The protein level of TRIM69 was also increased with DENV-2 infection in a virus dose-dependent manner (Fig 1B). Consistent with this, TRIM69 was also upregulated in A549, HUVEC and PBMC cells infected with DENV-2 (Fig 1C). In addition, the expression of TRIM69 was increased in peripheral blood cells from DENV-2-infected mice (Fig 1D). When HUVEC and HFF cells were stimulated with SeV, TRIM69 was also upregulated (Fig 1E).

A previous study reported the expression of TRIM family genes in response to interferons in immune cells. TRIM69 was identified as one of 27 TRIM genes which were induced by interferons [50]. Consistent with their results, we also found that the expression of TRIM69 mRNA and protein were induced in 293T, HUVEC and HFF cells upon IFN-*β* stimulation (Fig 1F and 1G). Four of other five selected ISGs, LGALS3BP, C19ORF66, DDX60, and HELZ2, were also induced in 293T cells stimulated with IFN-*β* (S2B Fig). Taken together, these data further confirmed that TRIM69 is an ISG induced by type I IFN and virus infection.

### DENV replication is restricted by TRIM69

**Fig 2:**
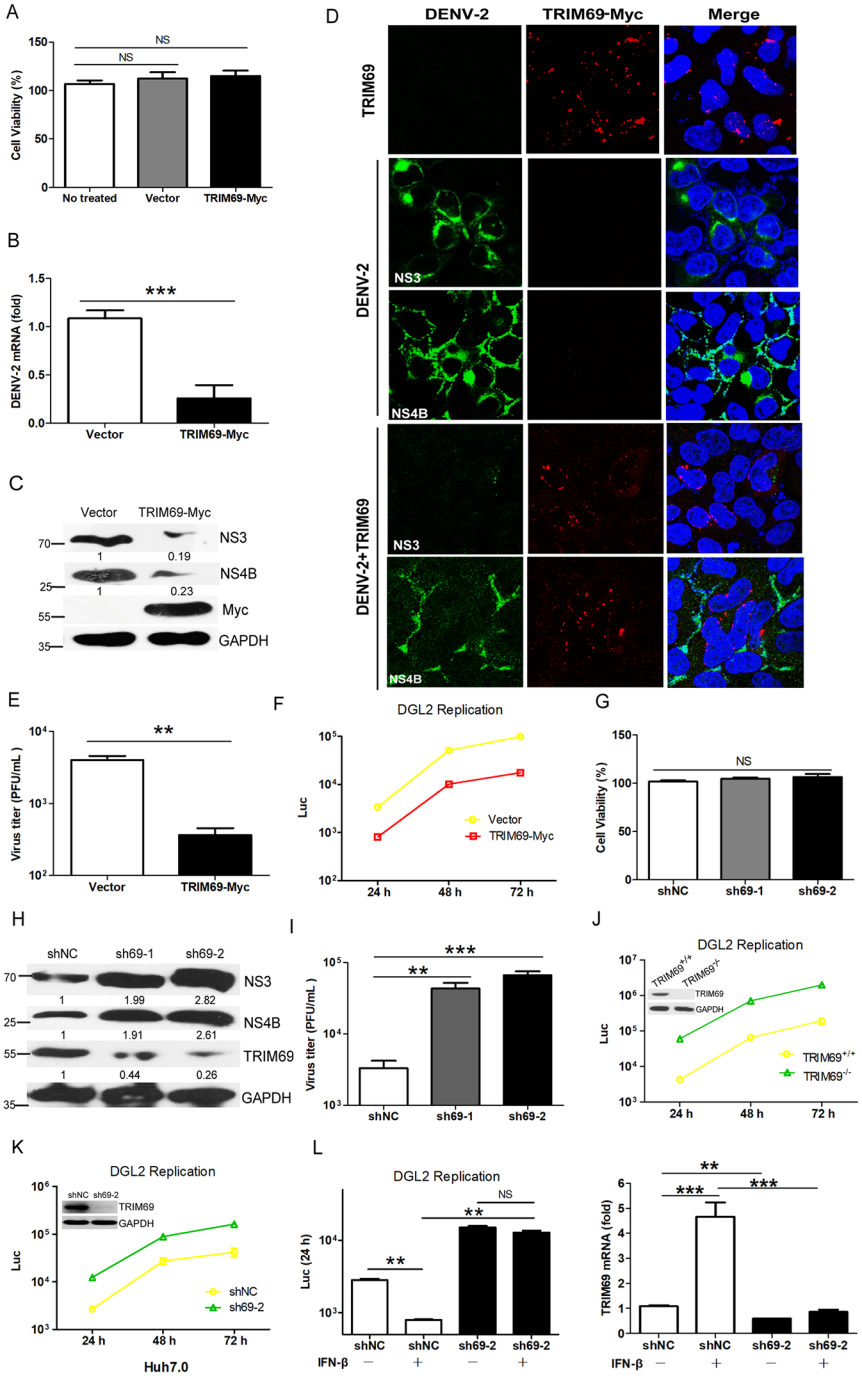
DENV replication is restricted by TRIM69. (A) Cell viability assays of transient transfections of TRIM69 or vector. (B-E) DENV replication was restricted by TRIM69 ectopic expression. DENV RNA (B) and NS proteins (C) expression were analyzed in DENV-infected 293T cells with or without TRIM69 overexpression. (D) IF analysis of DENV proteins in Hela cells. Hela cells were transfected with or without TRIM69-Myc for 24 h, then infected with DENV-2 for another 24 h. NS3/NS4B rabbit Ab and Myc mouse Ab were used to perform the IF assays. (E) The titers of DENV from supernatants of infected 293T cells with or without TRIM69-Flag, as determined by TCID_50_ assays. (F) DENV DGL2 replicon replication was inhibited by TRIM69 ectopic expression. The supernatants from the cells were collected at indicated time-points post transfection to test the luciferase activity. (G-I) DENV-2 infection of normal (shNC) or TRIM69 knockdown (sh69-1 and sh69-2) 293T cells. (G) TRIM69 knockdown by shRNA did not cause cell toxicity. (H) NS3 and NS4B expression in TRIM69 silenced or control cells as tested by western blot. (I) Virus titers of DENV-2 from the supernatants of normal or TRIM69 knockdown cells. (J) DGL2 replicon replication in TRIM69 knockout 293T cells. TRIM69^-/-^ cell line was generated by CRISPER/Cas9 system. (K) DGL2 replicon replication in Huh7.0 cells with TRIM69 knockdown. (L) The inhibitory efficiency of IFN-*β* on DGL-2 replication in normal or TRIM69 knockdown 293T cells (left). The TRIM69 mRNA levels in these cells were indicated by qRT-PCR (right). Results are expressed as mean ± SEM. * *p* < 0.05, ** *p* < 0.01, and *** *p* < 0.001. The data shown are representative of at least 3 independent experiments.

To explore the function of TRIM69 on DENV replication, cells were transfected with TRIM69-Myc and then infected with DENV-2. Transient overexpression of TRIM69 did not cause noticeable cell toxicity at 72 h post transfection (Fig 2A). qRT-PCR and Western Blot results suggested that the viral RNA and proteins were significantly decreased in TRIM69 overexpressed cells compared with control cells (Fig 2B and 2C). Immunofluorescence (IF) assay also confirmed that the viral NS3 and NS4B protein levels were significantly decreased in TRIM69 overexpressed cells (Fig 2D). Consistently, the released viruses in cell supernatants were also decreased in TRIM69 overexpressed cells (Fig 2E). In addition, we also used a luciferase-based DENV replicon (DGL2), derived from DENV-1 [51,52], to analyze the function of TRIM69 on virus replication. The replicon replication was also impaired by TRIM69 ectopic expression (Fig 2F).

To further confirm this phenotype, two TRIM69 knockdown shRNAs (sh69-1 and sh69-2) were constructed. Silencing TRIM69 by shRNA transfection did not influence cell viability (Fig 2G). The abundance of both DENV NS3 and NS4B proteins was significantly increased in two TRIM69 shRNAs transfected 293T cells after DENV-2 infection (Fig 2H). The virus titers in cell supernatants were also increased in TRIM69 silenced cells (Fig 2I). In addition, a TRIM69 knockout stable cell line was generated by CRISPER/Cas9 system, and the replication of DGL2 replicon was significantly increased in TRIM69 knockout cells compared with controls (Fig 2J). Furthermore, the replication of DGL2 was also increased in TRIM69 silenced Huh7.0 stable cell line (Fig 2K).

Since TRIM69 is induced by IFNs and plays a role to restrict DENV infection, we wondered whether TRIM69 is critical for the efficacy of IFN on DENV inhibition. Viral replication assays suggested that IFN-*β* treatment could not efficiently suppress DGL2 replication in TRIM69 silenced cells (Fig 2L). This demonstrates that TRIM69 is critical for IFN mediated anti-DENV activity.

To investigate whether mouse TRIM69 has the same function as its human homolog, mTRIM69 was overexpressed or silenced in mouse B16F10 cells. The results suggested that viral proteins expression and viral titers of DENV were significantly decreased in mTRIM69-myc transfected B16F10 cells compared with controls (S3A Fig). Furthermore, DENV infection was also obviously increased in mTRIM69 silenced cells (S3B and S3C Fig). Altogether, these data illustrate that both human and mouse TRIM69 protein acts as antiviral factors for DENV replication.

### The E3 ubiquitin ligase activity of TRIM69 is required for DENV restriction

TRIM69 is a TRIM family member containing a RING domain with E3 ubiquitin ligase activity. We next tested whether the E3 ubiquitin ligase activity of TRIM69 is necessary for DENV inhibition.

**Fig 3:**
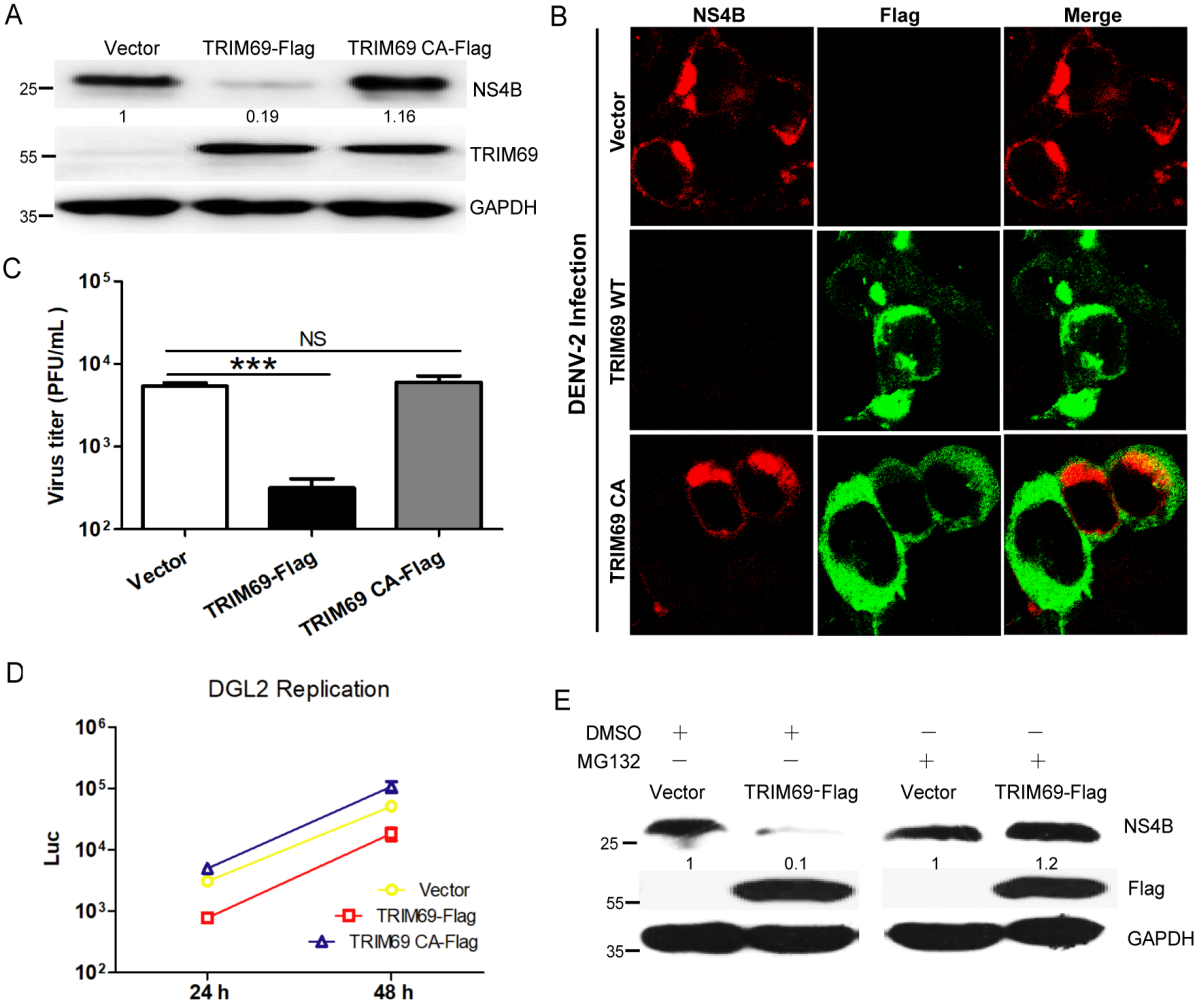
TRIM69 E3 ubiquitin ligase activity is required for DENV inhibition. (A) DENV-2 NS4B was decreased in 293T cell lines stably expressing TRIM69-Flag, but not TRIM69 CA-Flag. TRIM69, TRIM69 CA and control cell lines were infected with DENV-2 for 24 h, and the NS4B protein was analyzed by western blot (A) and IF assay (B). For IF assay, stable cell lines were infected with DENV-2 and stained with anti-NS4B and anti-Flag antibodies, respectively. (C) Virus titers were tested from the supernatants of control, TRIM69, and TRIM69 CA stable cell lines. (D) TRIM69 CA does not restrict DGL2 replicon replication. DGL2 was transfected into control, TRIM69, and TRIM69 CA stable cell lines, and the supernatants were harvested at the indicated time-points. (E) DENV-2 infection (as shown by protein level of NS4B) is reduced by TRIM69 ectopic expression, but recovered with MG132 treatment. Cells were infected with DENV-2 for 12 h and then treated with MG132 (2 μM) or DMSO for 12 h before harvested.

TRIM69 CA, a mutant TRIM69 with the catalytic amino acids Cys61 and Cys64 of the RING domain substituted by two Alanines, loses its E3 ubiquitin ligase activity [53]. Cell lines stably expressing TRIM69-Flag and TRIM69 CA-Flag were generated using pLV-Flag vector by antibiotics selection. Western Blots confirmed that stable cell lines expressed higher level of TRIM69 (or TRIM69 CA) compared with endogenous TRIM69 (Fig 3A). After DENV-2 infection, the abundance of viral NS4B, as shown by Western Blots (Fig 3A) and IF assay (Fig 3B), was decreased in TRIM69 expressing cell line, but not in TRIM69 CA cells. The virus titers from the cells stably expressing TRIM69 were lower than the control or TRIM69 CA (Fig 3C). In line with this, DGL2 replication was also impaired in TRIM69, but not TRIM69 CA, overexpressed cells (Fig 3D). When the cells treated with MG132, a proteasome inhibitor, the content of NS4B was recovered in TRIM69 overexpressed cell after DENV infection (Fig 3E). These results indicate that the E3 ubiquitin ligase activity of TRIM69 is critical for its antiviral activity.

### Knockdown of TRIM69 renders mice susceptible to DENV infection

**Fig 4:**
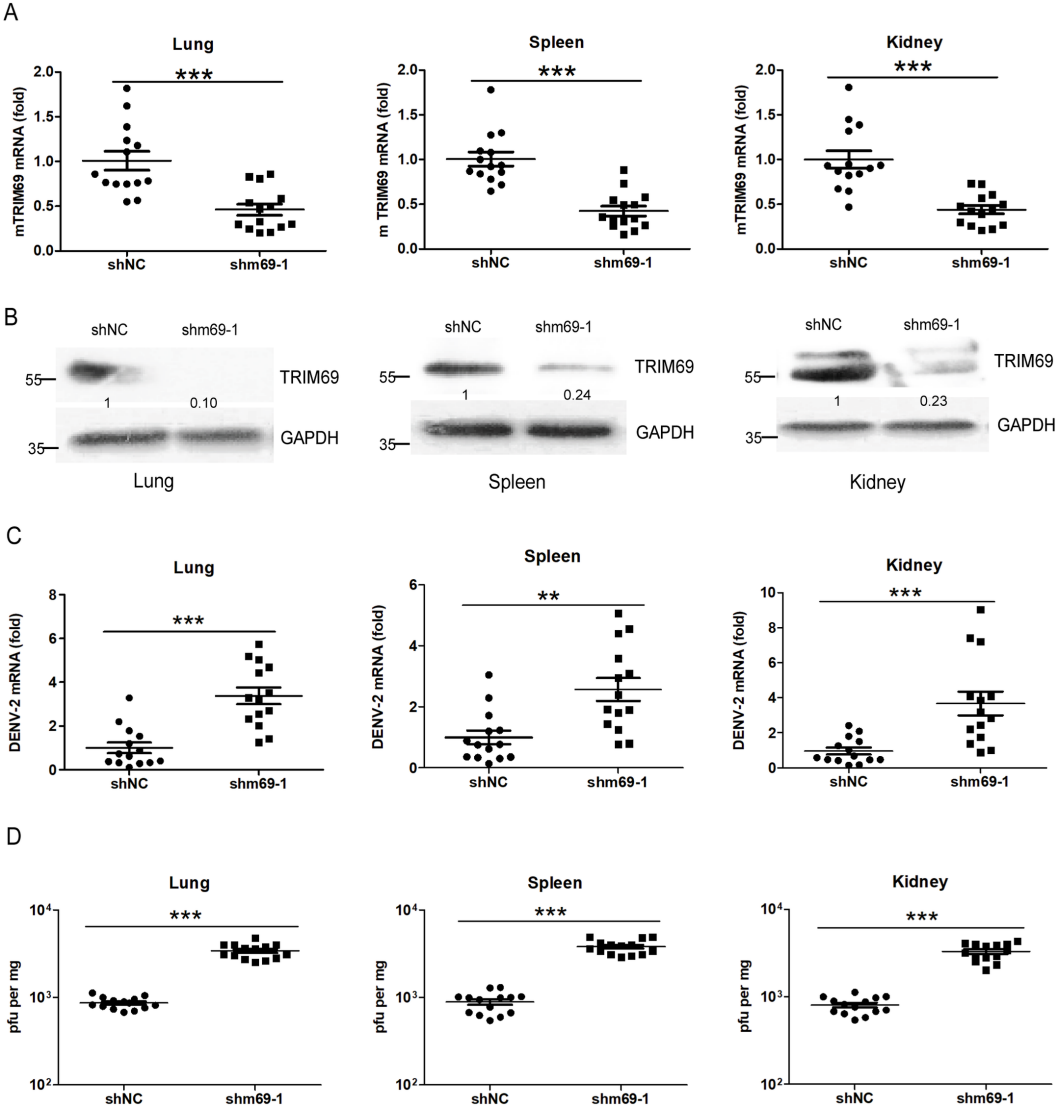
TRIM69 is a restrictor for DENV replication *in vivo*. For each experiment, 5×10^7^ *pfu* of lentiviral shm69-1 or shNC was injected into mice individually from caudal vein. 7 day post lentiviruses injection, mice were challenged with DENV-2 (1×10^7^ *pfu*) *via* intravenous injection for 3 days. The knockdown efficiency of shm69-1 in mouse lung, spleen and kidney were detected by qRT-PCR (A) and Western Blot (B). DENV infection in organs of TRIM69 silenced mice were measured viral RNA by qRT-PCR (C) or viral titers by TCID_50_ assay (D). Results are expressed as mean ± SEM. * *p* < 0.05, ** *p* < 0.01, and *** *p* < 0.001 (t-test). Data were pooled from three independent experiments.

Previous study suggested DENV causes a transient infection in immunocompetent mice with detectable virus in various organs [54]. To explore the function of TRIM69 on DENV *in vivo*, shm69-1 and shNC lentiviruses were generated and used to inject into mice *via* caudal vein. 7 days post lentivirus infection, mice were challenged with DENV-2 by intravenous injection. qRT-PCR and western blot suggested that mouse TRIM69 was silenced by shm69-1 lentiviruses in mouse lung, spleen and kidney (Fig 4A and 4B). In consistent with the *in vitro* data, both DENV RNA level (Fig 4C) and virus titers (Fig 4D) were significantly increased in organs from TRIM69-silenced mice. These data further confirmed that TRIM69 is an important host antiviral factor against DENV *in vivo*.

To test whether TRIM69 also restrict other virus infection, TRIM69 overexpressing or control cells were infected with influenza virus H1N1 (an RNA virus) or herpes virus HSV-1 (a DNA virus), respectively. The results suggested that TRIM69 did not interfere with H1N1 or HSV-1 infection (S4A and S4B Fig). TRIM69-silenced mice showed similar susceptibility with wide type mice to H1N1 infection (S4C and S4D Fig). These data suggest that TRIM69 may play a specific antiviral activity against DENV.

### DENV NS3 is specifically targeted by TRIM69

Several members of TRIM family proteins were reported to restrict viral replication by modulate the IFN pathways. We next tested whether TRIM69 is involved in IFN or ISG activation. Results suggested that overexpressing or silencing TRIM69 did not significantly influence SeV-induced IFN or ISG production (S5 Fig). This is also consistent with previous report by Versteeg G *et al*., that TRIM69 does not modulate either IFN production or ISG expression [33].

**Fig 5:**
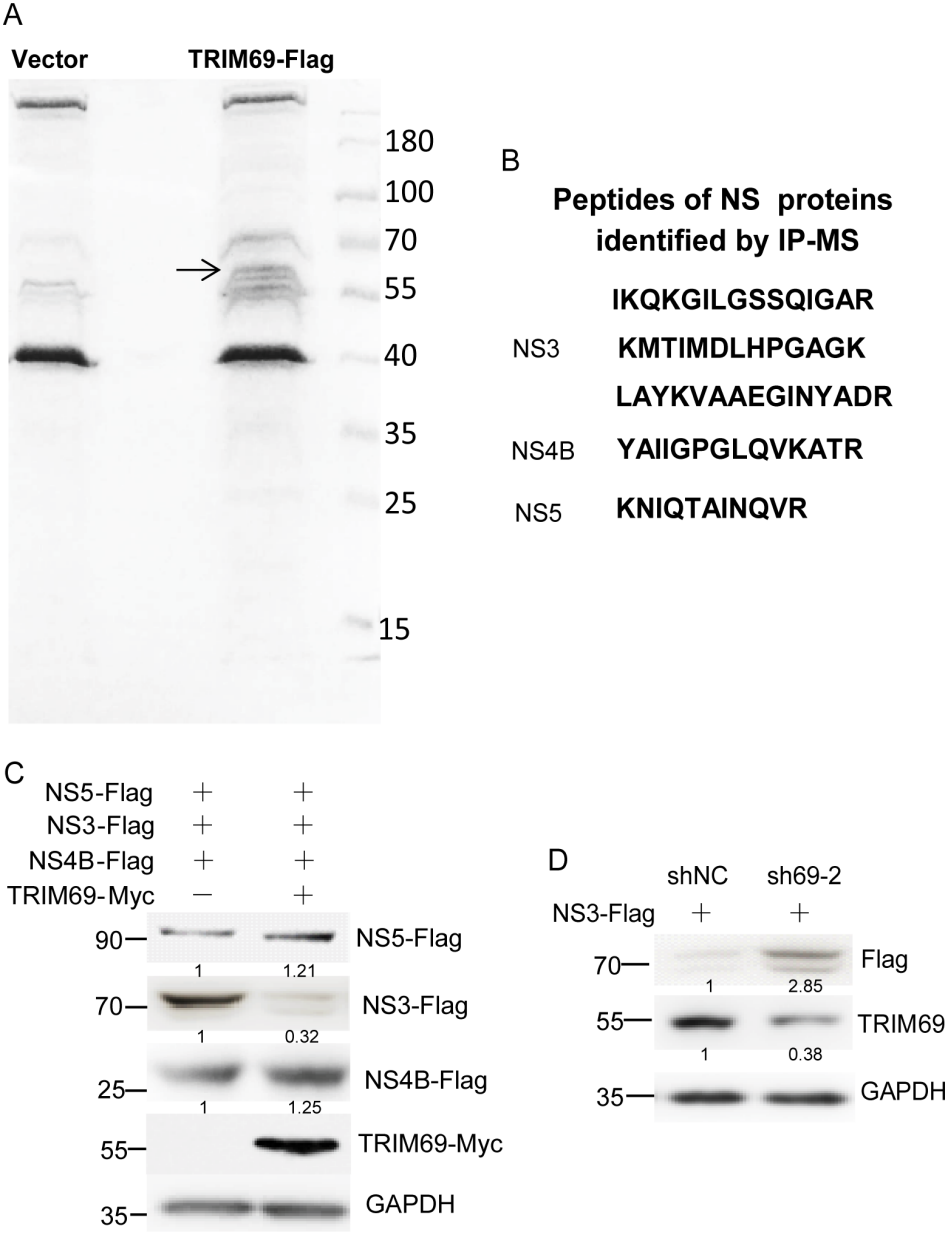
DENV NS3 is a target of TRIM69. (A) Proteins pulled down by Flag tag or TRIM69-Flag were loaded on SDS-PAGE with coomassie blue staining. The band of TRIM69 protein was indicated by an arrow. (B) Peptides of NS proteins identified by IP-MS. The two samples from Fig 5A were subjected to MS analysis. The peptides of NS proteins immunoprecipitated by TRIM69-Flag were shown. (C) NS3, but not NS4B or NS5, was degraded *via* TRIM69 ectopic expression. (D) NS3 expression was upregulated in TRIM69 silenced cells. (Eukaryotic expression of NS3 results in two bands in this study[65,71]. This is consistent with results described previously and the smaller protein may have arisen from internal initiation of translation).

To further elucidate the mechanisms of TRIM69 on DENV inhibition, immunoprecipitation and mass spectrometry (IP-MS) were performed to find out proteins that interact with TRIM69 during DENV infection (Fig 5A and S6A Fig). Three viral proteins, NS3, NS4B and NS5, were pulled down by TRIM69-Flag coupled beads but not with beads alone (S6B Fig). Three peptides of NS3, one peptide of NS4B, and one peptide of NS5 were identified by IP-MS (Fig 5B). These three viral proteins were co-expressed in 293T cells with or without TRIM69-Myc. The result suggested, the abundance of NS3, but not NS4B or NS5, was significantly reduced in TRIM69 overexpressed cell compared with controls (Fig 5C). Moreover, the ectopic expression of NS3 was increased *via* TRIM69 knockdown by sh69-2 (Fig 5D). This suggests that NS3 is a target for TRIM69.

NS3 forms a protease complex with NS2B, not only responsible for cleavage of viral polyprotein, but also for immune evasion [19,20]. NS2B3 could specifically cleave human STING, play a role to escape STING mediated antiviral pathway. We then tested whether TRIM69 also influences the cleavage activity of NS2B3 on STING. We found that TRIM69 significantly reduced the amount of NS2B3 protein, thereby impaired the cleavage of STING (S7A Fig). These data further suggest that TRIM69 targets NS3 and modulates NS3 function.

### TRIM69 interacts with DENV NS3

**Fig 6:**
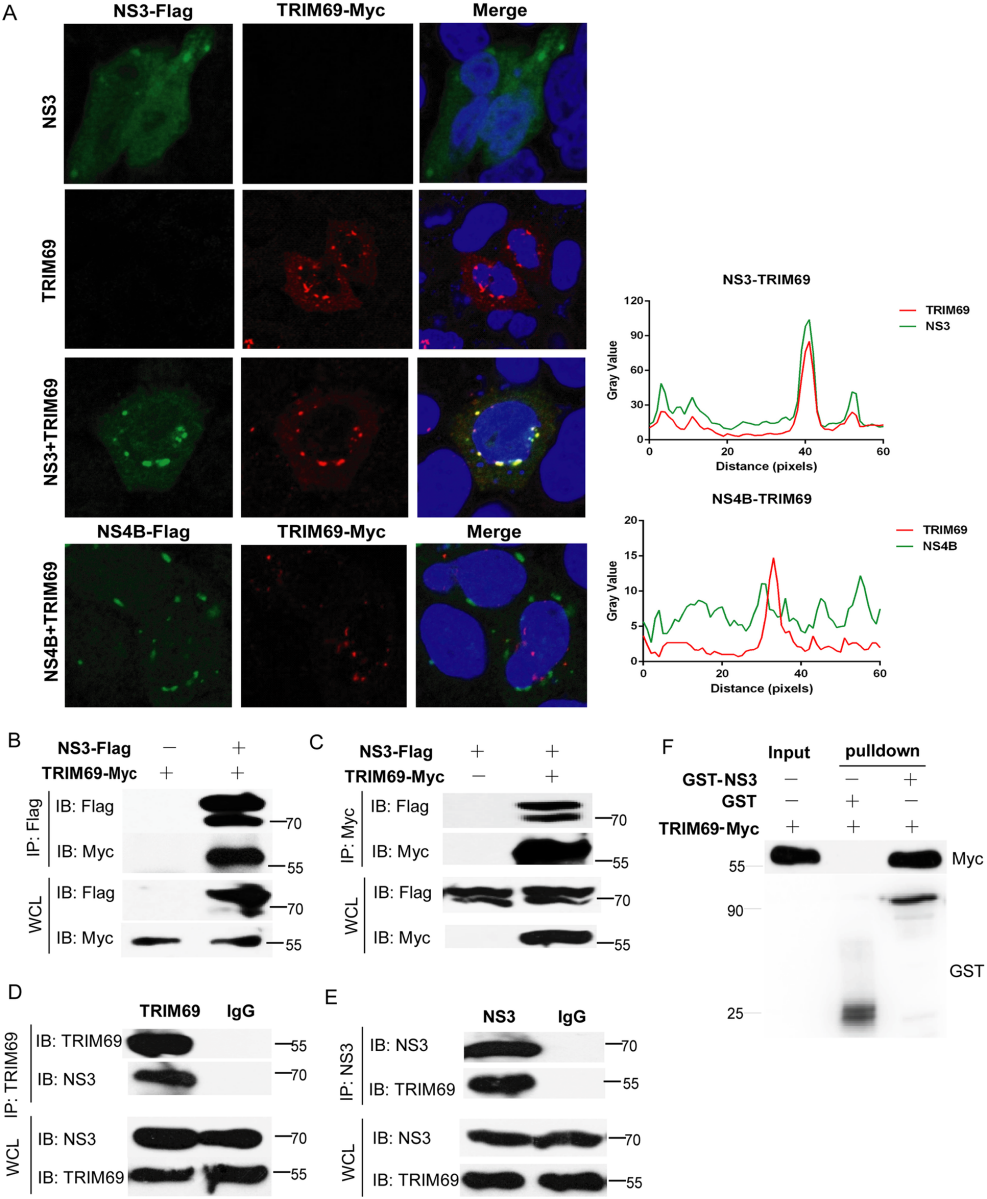
TRIM69 interacts with DENV NS3. (A) Confocal microscopy of TRIM69-Myc (red) and NS3-Flag or NS4B-Flag (green). NS3-Flag or NS4B-Flag was transfected into Hela cells with or without TRIM69-Myc for 48 h, and then the cells were treated with MG132 for 4 h before fixation. The nuclear was stained with DAPI. (B) Co-IP of lysates from 293T cells expressed TRIM69-Myc with or without NS3-Flag. IP was performed using Flag antibody. (C) Reciprocal Co-IP of lysates from 293T cells transfected with NS3-Flag with or without TRIM69-Myc. IP was performed using Myc antibody. (D, E) Co-IP of endogenous TRIM69 and NS3 from lysates of 293T cells infected with DENV-2 for 48 h. IP was performed with TRIM69 antibody (D) or NS3 antibody (E). IgG was used as a control. (F) GST-pulldown assays of GST-NS3 with purified TRIM69-Myc. Representative blots from three different repeats were shown.

To confirm the interaction between DENV NS3 and TRIM69, the cellular distribution of NS3 and TRIM69 was examined by confocal microscopy. When co-expressed with TRIM69, NS3 is re-distributed from a predominantly diffuse cytoplasmic localization to punctate sites co-localizing with TRIM69 (Fig 6A). The co-localization of TRIM69 and NS3 was specific, as another viral protein, NS4B, did not co-localize with TRIM69 (Fig 6A). Co-IP assays were performed to further confirm the physical interaction between TRIM69 and NS3. IP of NS3 with Flag antibody successfully coprecipitated TRIM69-Myc (Fig 6B). Likewise, the reciprocal test using Myc antibody could immunoprecipitate TRIM69 with NS3 (Fig 6C). Furthermore, endogenous TRIM69 also interacted with NS3 (Fig 6D and 6E) from DENV infected cells. Finally, a GST pulldown assay also confirmed that purified TRIM69 protein interacts with GST-NS3 directly (Fig 6F).

The interaction between mTRIM69 and NS3 was also investigated in mouse cells. mTRIM69 also co-localized and interacted with NS3 in B16F10 cells (S8A and S8B Fig). These data suggest that TRIM69 interacts with DENV NS3.

### TRIM69 is an ubiquitin ligase of DENV NS3

**Fig 7:**
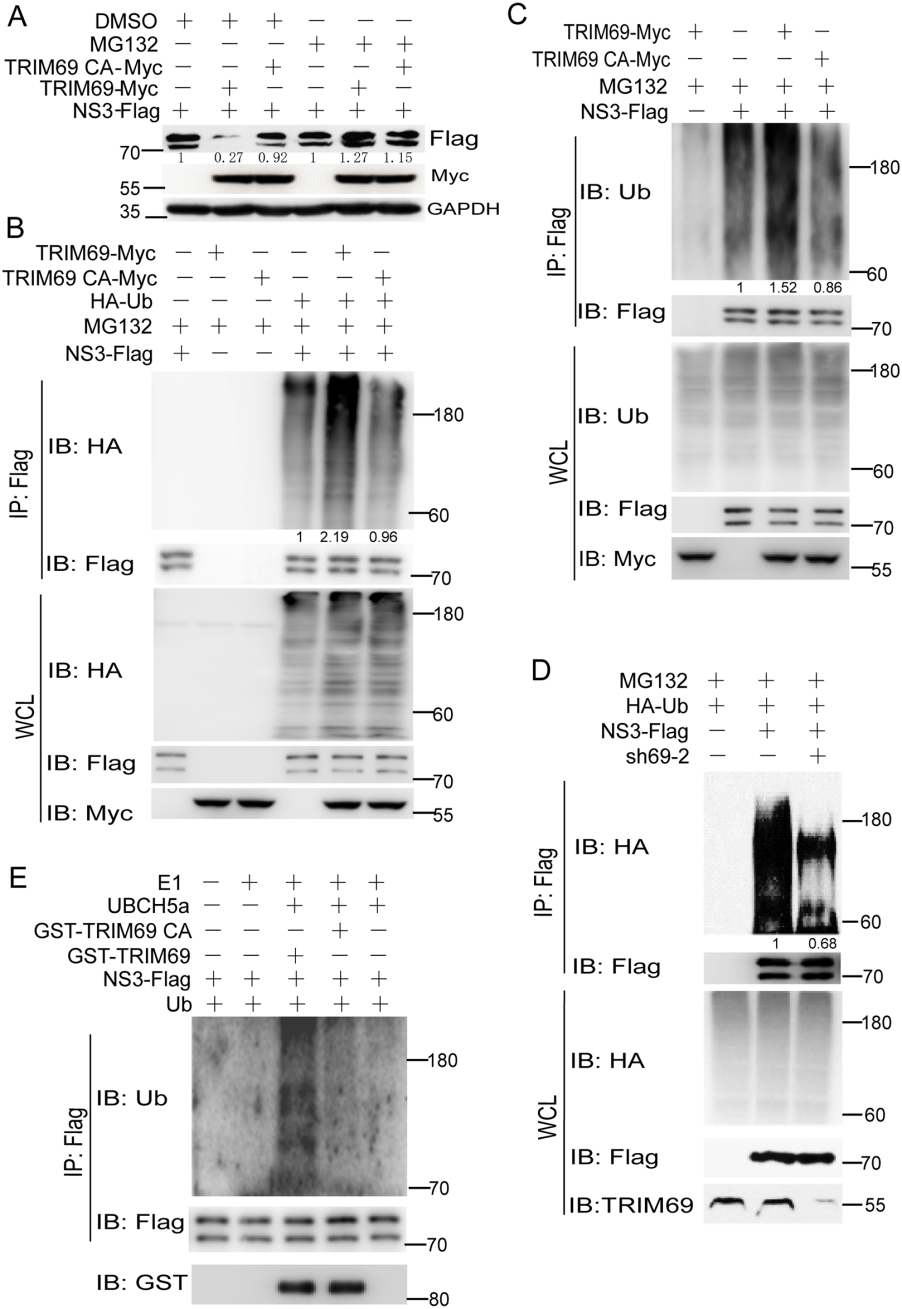
DENV NS3 is degraded *via* TRIM69 ubiquitination. (A) The degradation of NS3 by TRIM69 was recovered by MG132. NS3-Flag was transfected into 293T cells with or without TRIM69-Myc for 48 h, and treated with MG132 for 4h as indicated. (B, C) NS3 ubiquitination was increased when co-transfected with TRIM69, but not with TRIM69 CA. NS3-Flag and TRIM69-Myc (or TRIM69 CA-Myc) were co-transfected into 293T cells together with (B) or without (C) HA-Ub for 48 h. NS3 was immunoprecipitated with Flag antibody, and ubiquitination of NS3 was detected with HA (for Ub-HA) (B) or Ub (C) antibody. (D) The ubiquitination of NS3 was decreased in TRIM69 silenced 293T cells. NS3-Flag and HA-Ub were co-transfected into 293T cells together with shNC or sh69-2 for 48 h. (E) *In vitro* ubiquitination of NS3 by TRIM69. *In vitro* ubiquitination assay was performed using an E3 Ligase Auto-Ubiquitination Assay Kit (Abcam) according to manufacturer’s instructions. Representative blots from three different repeats were shown.

Since TRIM69 is an E3 ligase, we next investigated whether NS3 is ubiquitinated by TRIM69. As shown in Fig 7A, overexpressing TRIM69, but not TRIM69 CA, led to NS3 degradation; however, this degradation was blocked by MG132. When the ectopically expressed NS3 was immunoprecipitated by Flag, we observed ubiquitination modifications on NS3, and the ubiquitination of NS3 was obviously increased in the presence of TRIM69-Myc, but not of TRIM69 CA-Myc (Fig 7B). We also detected more endogenous ubiquitin conjugated to NS3 in the presence of TRIM69, but not TRIM69-CA (Fig 7C). Consistent with this, the ubiquitin ligated to NS3 was significantly reduced when TRIM69 was knockdown (Fig 7D). Finally, an *in vitro* ubiquitination assay further confirmed that TRIM69 can directly ubiquitinate NS3 in the presence of ubiquitin E1 and E2 in a cell-free system (Fig 7E).

### Lys104 of NS3 is an ubiquitination site for TRIM69

**Fig 8:**
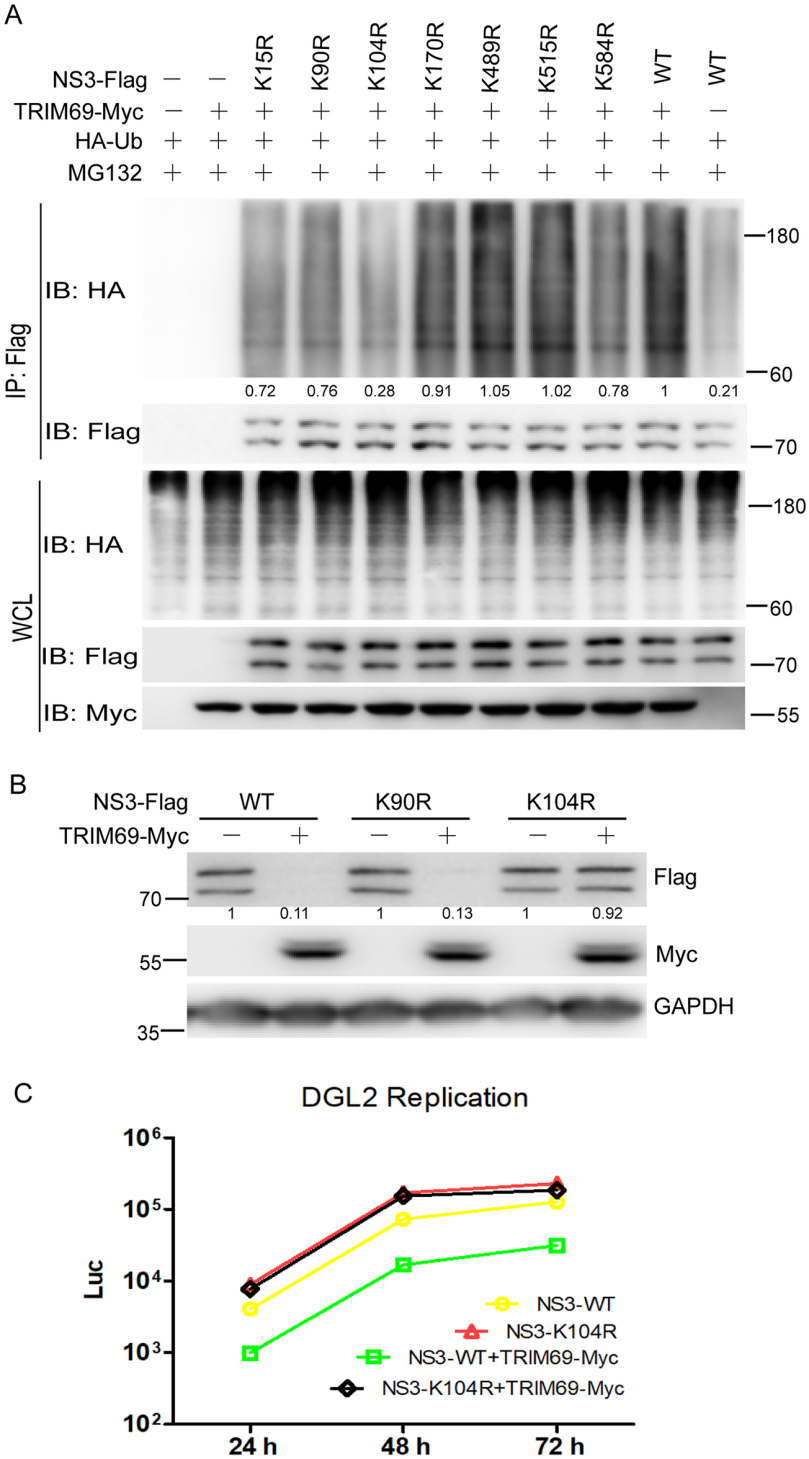
Lys104 of NS3 is a target of TRIM69-mediated ubiquitination. (A) Immunoblot analysis of the ubiquitination of wildtype or mutants NS3 in 293T cells. Flag tagged NS3 (or NS3 mutants) and HA-Ub were co-transfected into 293T cells individually in the presence of ectopic expressed TRIM69-Myc. Immunoprecipitation was performed with Flag antibody. The expressions of indicated protein were shown at bottom panels. (B) NS3 and two NS3 mutants (K90R and K104R) were transfected with or without TRIM69-Myc for 48 h. The degradation of NS3 was visualized by western blots. (C) DGL2 NS3-WT and NS3-K104R were transfected into 293T cells together with or without TRIM69-Myc. The supernatants from the cells were harvested at indicated time-points to detect the luciferase activity. Results are expressed as mean ± SEM. * *p* < 0.05, ** *p* < 0.01, and *** *p* < 0.001. The data shown are representative of at least 3 independent experiments.

DENV-2 NS3 contains 46 lysine residues. Seven (Lys15, Lys90, Lys104, Lys170, Lys489, Lys515, and Lys584) of these were predicted to be potential ubiquitination sites by the UbPred program (http://www.ubpred.org/). To determine the NS3 ubiquitination sites by TRIM69, we replaced each of the seven NS3 lysine residues noted above individually with arginine. Immunoprecipitation with anti-Flag and immunoblot analysis of ubiquitin demonstrated that K104R substitution significantly decreased the ubiquitination of NS3 by ectopically expressed TRIM69 (Fig 8A). Furthermore, NS3 WT, K90R, and K104R were transfected into 293T cells together with or without TRIM69-Myc. The immunoblot analysis showed that the expression of NS3 WT and K90R were significantly reduced *via* TRIM69 ectopic expression, however, K104R was not (Fig 8B).

To further confirm the Lys104 is a potential ubiquitination site of NS3 for TRIM69, we constructed a mutant DENV-1 DGL2 replicon (NS3-K104R) *via* site-directed mutagenesis, in which the Lys104 of NS3 being replaced by arginine. DGL2 NS3-WT and NS3-K104R were transfected into 293T cells individually together with or without TRIM69-Myc. The results revealed that the replication of DGL2 reduced with TRIM69 ectopic expression, however, the replication of NS3-K104R did not (Fig 8C). All the data illuminate that Lys104 is an ubiquitination site of NS3 for TRIM69.

## Discussion

This work has illustrated a TRIM family member, TRIM69, as a key host factor needed to restrict DENV infection. TRIM69 mRNA was found to be overexpressed in 293T cells infected with DENV-2 as determined by RNA-Seq analysis. A significant amount of differential expressed genes found in virus infected cells have been described as signaling pathway molecules involved in antiviral innate immunity (S1 Fig). 52 out of the 99 upregulated genes are predicted ISGs, such as TRIM69, LGALS3BP, C19ORF66, DDX60, and HELZ2 (S2 Fig). All of these putative ISGs were upregulated after DENV infection (S2 Fig). Consistent with our results, two recent studies also report that both C19ORF66 and HELZ2 are induced by IFN and suppress DENV replication [55,56]. A previous report identified that TRIM69 is induced in peripheral blood cells upon type I IFN stimulation [50], here we show that TRIM69 is also upregulated in 293T, HUVEC, and HFF cells by IFN-*β* stimulation or virus infection (Fig 1), and has antiviral properties against DENV infection.

Many TRIM family proteins are involved in regulating signaling pathways such as Toll-like receptors (TLRs) and RIG-I-like receptors (RLRs) which are needed for viral detection and innate immune responses [57]. For example, TRIM12c interacts with TRAF6, leading to a cooperative activation of IFN and NF-κB pathways [58]. TRIM38 negatively regulates TLR3/4-mediated innate immune and inflammatory responses [59]. TRIM13 acts as a negative regulator for MDA5-mediated type I interferon production [60]. Since TRIM69 is an ISG, we also tested whether or not it participates in IFN-induced signal pathway. Unlike other reported TRIM family members, TRIM69 did not influence SeV-induced IFN-*β* production or IFN-*β*/SeV induced ISRE promoter activation, which were consistent with the findings from a previous screening [33]. They found roughly half of the 75 TRIM family members modulated the interferon response, but TRIM69 was not in the list [33]. These results suggest that TRIM69 has no influence on IFN production or IFN function. In line with this, we also found that TRIM69 did not influence other virus infection, such as H1N1 or HSV-1 (S4 Fig). These results suggest that TRIM69 may use a specific mechanism to restrict DENV infection, independent of interferon pathway.

Some TRIM proteins have been demonstrated to have direct antiviral activity, including TRIM5α, TRIM22, and TRIM79α [41,44,61–64]. To further investigate the mechanism of TRIM69 on inhibiting DENV replication, IP-MS analysis was performed to search for host and viral proteins interacting with TRIM69. DENV NS3 was found directly interacts with TRIM69 and degraded *via* TRIM69 ectopic expression (Fig 5). Then, we tested whether TRIM69 also influences the function of NS3. A recent study suggested that a K27-linked ubiquitination of NS3 enhance the interaction of NS3 and NS2B, thereby promotes the cleavage STING by NS2B3 complex[65]. We found that overexpression of TRIM69 impaired the cleavage of STING by NS2B3 (S7A Fig). This is reasonable, since TRIM69 targets NS3 to degradation, NS2B3 level will also be decreased (S7A Fig). While, we found TRIM69 seems not influence the interaction efficiency of NS2B and NS3 (S7B Fig). A possible reason is that our other preliminary experiments suggest TRIM69 may influence K11-linked ubiquitination of NS3, rather than previously reported K27-linked ubiquitination [65]. And K11-linked poly ubiquitination can mediates protein degradation in a proteasome dependent manner [66]. Further experiments will be required to address the detailed ubiquitination form on NS3 mediated by TRIM69.

In this study we found, TRIM69 acts as an IFN-*β*-stimulated ISG and has antiviral activity *via* its RING domain. As an E3 ubiquitin ligase, TRIM69 was reported to restrict DENV replication by direct ubiquitination of NS3 which leads to NS3 degradation. The viral protease NS3 is highly conserved throughout the *Flavivirus* genus and necessary for viral replication and immune evasion [67,68]. We next will further investigate whether TRIM69 acts as a broad-spectrum restriction factor for all the closely related mosquito-borne flaviviruses.

## Methods and materials

### Ethics statements

The HUVEC (Human Umbilical Vascular Endothelium Cells) and PBMC (human Peripheral Blood Mononuclear Cells) were obtained from BeNa Culture Collection (Bejing, China). All samples were anonymized and the projects using of human biological specimens were approved by an institutional review board (IRB) of Soochow University.

Animal experiments were conducted according to the Guide for the Care and Use of Medical Laboratory Animals (Ministry of Health, People’s Republic of China) and approved by the Animal Care and Use Committee as well as the Ethical Committee of Soochow University (SYSK-(S2012-0062)).

### Cells and viruses

293T, Vero, HeLa, A549, Huh7.0, and HFF cells were obtained from ATCC (Manassas, USA) and grown in DMEM (Life Technologies, Grand Island, USA) supplemented with 10% FBS and antibiotics/antimycotics. HUVEC, PBMC and B16F10 cells were grown in 1640 (Life Technologies) supplemented with 10% FBS and antibiotics/antimycotics.

DENV type 2 (DENV-2) New Guinea C (NGC) strain was propagated in mosquito C6/36 cells (ATCC CRL-1660). Cells were infected with DENV at a multiplicity of infection (MOI) of 1, unless otherwise stated. Influenza A virus (H1N1-A/PR/8/34) and Sendai virus (SeV) was propagated in 10 days old embryonated eggs (Bejing Laboratory Animal Research Center, Beijing, China), and the virus titer was detected by hemagglutination assay using chicken red blood cells (BeNa Culture Collection (Beijing, China)). Human herpesvirus 1 (HSV-1) was propagated in Vero cells.

### Antibodies and reagents

The following antibodies were purchased from Cell Signaling Technology (CST, Danvers, USA), rabbit anti GAPDH (Cat # 2118), mouse anti Flag (Cat # 8146), mouse anti HA (Cat # 2367) and anti-rabbit IgG HRP-linked Antibody (Cat # 7074). Others were obtained as follows; mouse anti Myc (Cat # M20002 Abmart, Shanghai, China), anti-Flag M2 Affinity Gel (Cat # A2220 Sigma-Aldrich, St Louis, USA), rabbit polyclonal anti DENV2 NS3 (Cat # PA5-32199 Thermo Fisher Scientific, Waltham, USA), rabbit polyclonal anti DENV2 NS4B (Cat # GTX113374 GeneTeX, Irvine, USA), rabbit polyclonal anti ZIKV NS3 (Cat # GTX133309 GeneTeX, Irvine, USA), rabbit anti TRIM69 (RNF36, Cat # ab111943 Abcam, Cambridge, UK), and HRP Goat anti-mouse IgG (Cat # 405306 Biolegend, San Diego, USA).

Lipofectamine 2000 was purchased from Life Technologies. MG132, puromycin, and dimethylsulfoxide (DMSO) were purchased from Sigma-Aldrich. N-ethylmaleimide (NEM) was obtained from Thermo Fisher Scientific. Protease inhibitor (PI) was from CST. Recombinant human IFN-β was from PeproTech (Rocky Hill, USA).

### Plasmids

TRIM69-Flag and TRIM69 CA-Flag were kindly provided by Dr. Linfang Wang (Tsinghua University, Beijing, China) [53]. To construct TRIM69-Myc, TRIM69 ORF was amplified from TRIM69-Flag and cloned into the *Xho* I and *EcoR* I sites of the pCMV-Myc vector. TRIM69 and TRIM69CA ORFs were also constructed into a puromycin resistant vector pLV-Flag for stable cell-line construction. To construct sh69-1 and sh69-2, target sequences (5’-GGGAAACTGATCTGCTTTC-3’ or 5’-GGACAAGTTGGTAGAGAAG-3’) were inserted into vector RNAi-Ready pSIREN-RetroQ-ZsGreen and Lenti-U6-shRNA-GFP-puro individually. To construct shm69-1 and shm69-2, target sequences (5’-GCTCGTGGAGAAGATTAAGAA-3’ or 5’-CGTTTCTTTACGGAGGAGCTT-3’) were inserted into vector Lenti-U6-shRNA-GFP-puro individually. For construction of CRISPR/Cas9 construct of TRIM69, the gRNA sequence (5’-GGCTCAAGAGGCTTCACCTC-3’) was cloned into pX462. All NS genes were amplified from cDNA of DENV-2 NGC strain. A DNA-based DENV replicon DGL2 (for DENV type 1) was generously provided by Dr. Takayuki Hishiki (Kyoto University, Kyoto, Japan) [51].

### Construction of stable cell lines

293T cells were transfected with pLV-TRIM69-Flag or pLV-TRIM69CA-Flag, and selected with puromycin (2 μg/mL) for at least 3 weeks. The overexpressions of TRIM69 and TRIM69CA in selected stable cell lines were confirmed by Western Blot. Similarly, shTRIM69 (U6-shRNA-GFP-puro) and TRIM69^-/-^ cell line (pX462) were constructed by puromycin selection followed by single cell clone culture and Western Blot identification.

### RNA-Seq and data analysis

Total RNA was collected from DENV-2 infected (or non-infected) 293T cells at 48h post-infection. cDNA libraries were prepared through the sequential use of the RNeasy Mini Kit with On-Column DNase Digestion Set (QIAGEN, Venlo, Netherlands), Dynabeads mRNA DIRECT purification Kit and Total RNA-Seq Kit v2 (Thermo Fisher Scientific). The transcription sequences were sequenced using an Illumina Hiseq2000, and the total base number was more than 20 Gb per sample. RNA-Seq de novo assembly was performed using Trinity. Get ORF in EBOSS were used to find protein from contigs.

### Dual-Luciferase Reporter (DLR) assays

100 ng expression plasmid, 50 ng IFN-β-Luc/ISRE-Luc, and 10 ng pRL-TK (internal control) were co-transfected into 293T cells plated in 96-well plates. Then the cells were treated with SeV infection or IFN-*β* (200 U/ml) stimulation where indicated. 24 hours later, cells were harvested and the DLR assays were performed with a luciferase assay kit (Promega, Madison, WI). All reporter assays were completed at least in triplicate, and the results were shown as average values ±standard deviations (SD) from one representative experiment.

### DENV replicon Gaussia luciferase reporter assay

In 96-well plates, 50 ng of DGL2 replicon plasmid was transfected into 293T cells stably expressing TRIM69 (or TRIM69 CA) or TRIM69 silenced (shTRIM69 or TRIM69^-/-^) cells. For the Gaussia luciferase assay, culture supernatants were collected at different time points and luciferase was measured using BioLux Gaussia Luciferase Assay Kit (New England Biolabs) according to manufacturer’s instructions.

### Immunofluorescence (IF) analysis

Hela cells were transfected with or without the plasmid TRIM69-Myc. The cells were then infected with DENV-2 for 24 h. Cells on coverslips were fixed in 4% formaldehyde for 10 min, sequentially permeabilized with 0.4% Triton X-100, blocked with 5% FBS, incubated with primary antibodies (rabbit anti-NS3, rabbit anti-NS4B and mouse anti-Myc) at 4 °C overnight, and incubated with TRITC-Goat anti-mouse IgG (H+L) (Jackson, Cat # 115-025-003) and Goat anti-Rabbit IgG-FITC (Southern Biotech, Cat # 4030–02) for 1 h at room temperature. Nuclei were counter stained with DAPI (0.5μg/ml). Finally, the images were obtained by confocal microscopy.

For co-localization study, plasmids with NS3-Flag or NS4B-Flag were transfected into Hela cells together with or without hTRIM69-Myc. To investigate the co-localization of mTRIM69 and NS3, NS3-Flag was transfected into mouse B16F10 cells together with or without mTRIM69-Myc. All the cells were treated with MG132 (20 μM) for 4 h before fixation.

### RNA isolation and qRT-PCR

Total RNA from the indicated cells treated by different treatments were extracted using the total RNA kit I (OMEGA) and reverse-transcribed using the PrimeScript Master Mix kit (TaKaRa). The resulting cDNAs were mixed with RT-PCR primers and SYBR Premix Ex Taq II (TaKaRa) and amplified for 40 cycles (95 °C 15 s, 60 °C 30 s, and 72 °C 15s). The qPCR primers for human TRIM69, and human *β*-actin were listed below: TRIM69 Forward: 5’-TCTGTGGGGCAGTCTAAGGA-3’, Reverse: 5’-CCATGGACACATGTTGCTGC-3’; and *β*-actin Forward: 5’-GGGCATGGAGTCCTGTGGCA, Reverse: 5’-GGGTGCCAGGGCAGTGATCTC-3’. The qPCR primers for mouse Trim69, and mouse *β*-actin were listed below: Trim69 Forward: 5’-GAGGAGATGGAGGTGAATC-3’, Reverse: 5’-TTGTGATGTCTGTGAGGAA-3’; and *β*-actin Forward: 5’-CGTTGACATCCGTAAAGAC-3’, Reverse: 5’-GAGCCAGAGCAGTAATCT-3’. All the qPCR results are represented as relative fold changes after normalized to *β*-actin controls.

### Virus titration

The titers of DENV-2 in cell-free supernatants were determined with a median tissue culture infective dose (TCID_50_) assay according to standard protocols on Vero cells [69]. Briefly, Samples were serially diluted and inoculated into Vero cells in 96-well plates. After 5-day incubation, cells were examined for cytopathic effects (CPE) under a light microscope. The virus titer (TCID_50_/ml) was calculated using the Reed-Muench method. 1 TCID_50_/ml was equivalent to 0.69 *pfu*/ml [69,70].

### Immunoprecipitation (IP) assays

TRIM69-Flag plasmid was transfected into 293T cells and then infected with DENV-2. The cells were treated with MG132 (20 μM) for 4 h before lysed with RIPA buffer (25 mM Tris•HCl pH 7.4, 150 mM NaCl, 1% NP-40, 1 mM EDTA, 5% glycerol) together with Protease Inhibitors (CST). Samples were centrifugated for 10 min to remove cellular debris. The lysates were incubated with Flag Ab conjugated agarose beads (Sigma-Aldrich) overnight at 4 °C. After immunoprecipitation, proteins were separated on SDS–PAGE gels (Invitrogen) and stained with coomassie blue staining. Gel slices were excised and proteins were reduced with 10 mM DTT prior to alkylation with 55 mM iodoacetamide. Peptides were extracted and analyzed by nano-LC-MS/MS (ekspertnanoLC, TripleTOF 5600-plus, AB Sciex, USA).

For co-immunoprecipitation (Co-IP) assays, NS3-Flag was transfected together with or without human or mouse TRIM69-Myc constructs. The lysate was incubated with Myc Ab overnight at 4 °C. Then protein A/G was added into the lysate and incubated for 4 hours. The beads were then washed four times and western blot analysis was performed to detect NS3 and TRIM69. TRIM69 antibody and NS3 antibody were used to in the Co-IP of endogenous TRIM69 and DENV NS3.

For GST pulldown assay, recombinant GST-NS3 and GST control were incubated with immunoprecipitation purified TRIM69-Myc protein. The proteins pulled out by GST agarose were analyzed by western blots.

### Ubiquitination assays

NS3-Flag and HA-Ub were co-transfected into 293T cells together with or without TRIM69-Myc. The cells were then treated with MG132 (20 μM) for 4h and lysed by RIPA buffer with PI and NEM. All the samples were heated at 95 °C for 5 min prior to affinity purification in 1% SDS to remove NS3 interacting proteins. Then the Flag Ab conjugated agarose beads were added into the samples separately. Following incubation overnight at 4 °C, the samples were examined *via* western blotting.

*In vitro* ubiquitination assay was performed using an E3 Ligase Auto-Ubiquitylation Assay Kit (Abcam) according to manufacturer’s instructions. Briefly, immunoprecipitated NS3 were incubated with purified recombinant TRIM69 (or TRIM69 CA), E1 (Hdm2), and E2 (UbcH5a) in the presence of ATP. The *in vitro* ubiquitination of NS3 was analyzed by western blots.

### DENV infection of TRIM69 lentiviruses treated mice

The lentiviral shRNA against mouse TRIM69 and matched control lentiviral vector were transfected into 293T cells together with the relative packaging plasmids. Lentiviruses were produced from the cells after 72 h transfection, and purified by ultracentrifugation. Then the 5×10^7^ *pfu* of lentiviruses derived from shm69-1 were injected into mice caudal vein. 7 day post lentiviruses injection, mice were challenged with DENV-2 (1×10^7^ *pfu*) *via* intravenous injection. 3 days post DENV infection, mice were sacrificed and the Lung, Spleen and Kidney organs were dissected to monitor the DENV replication.

### Statistical analysis

Prism 7 software (GraphPad Software) was used for charts and statistical analyses. The significance of results was analyzed by an unpaired two-tailed ANOVA test or Student’s *t*-test with a cutoff P value of 0.05.

## Supporting information

S1 Fig Expressions of molecules from antiviral signal pathway are changed after DENV infection in 293T cells by RNA-Seq analysis. (A) Color intensity refers to the mean relative expression fold changes in comparison to non-infected cells. Red for upregulated, green for down regulated, and black for no change. (B) Fold change and p value of selected genes listed in panel A. (inf: infinity)
(TIF)

S2 Fig mRNA expression of 5 selected genes treated by DENV-2 or IFN-*β*. qTR-PCR analysis of 5 selected genes stimulated with DENV-2 (A) or IFN-*β* (B) in 293T cells. Results are expressed as mean ± SEM. NS, not significant.* *p* < 0.05, ** *p* < 0.01, and *** *p* < 0.001. The data shown are representative of at least 3 independent experiments.
(TIF)

S3 Fig DENV replication was restricted by TRIM69 in mouse cells. (A) mTRIM69-Myc was transfected into mouse B16F10 cells for 24 h. Then DENV-2 was infected the cells for another 24 h. Cell lysates and supernatants were harvested for Western blot and TCID_50_ assays, respectively. (B) The knockdown efficiency of two shRNAs (shm69-1 and shm69-2) targeting mouse TRIM69 was detected in B16F10 cells. The mRNA level (left) and protein (right) of mouse TRIM69 were analyzed. (C) The viral proteins and virus titers were tested in mTRIM69 silenced B16F10 cells.
(TIF)

S4 Fig TRIM69 does not restrict H1N1 and HSV-1 infection. (A) H1N1 and HSV-1 nucleotide copies were comparable in TRIM69 overexpressed cells and control cells. (B) Viral titers of H1N1 and HSV-1 from supernatants of control or TRIM69 overexpressed cells. (C) Viral load of H1N1 in peripheral blood cells in control and TRIM69 silenced mice as determined by qRT-PCR (left). TRIM69 knockdown efficiency in peripheral blood cells were confirmed by qRT-PCR (right). NS, not significant. The data shown are representative of 3 independent experiments. (D) Survival curve of H1N1 infected wide type and TRIM69 silenced mice (n = 5). Mice were infected with intranasal infection of 2x10^5^ *pfu* H1N1and monitored daily for survival rates.
(TIF)

S5 Fig TRIM69 is not involved in IFN signal pathway. (A) TRIM69 or TRIM69 CA did not affect SeV-stimulated IFN-*β* activation. IFN-*β*-driven luciferase activity was determined by a dual-luciferase assay. (B) The RNA level of IFN-*β* was detected in TRIM69 transfected 293T cells stimulated with SeV. (C) TRIM69 overexpression did not influence IFN-*β*-stimulated ISRE promoter activation. 293T were treated with IFN-*β* for 12 h and harvested to test the ISRE-luciferase activity. (D) Knockdown of TRIM69 did not influence IFN-*β* or SeV-stimulated ISRE promoter activity. shNC or sh69-2 was co-transfected with ISRE-luc and pRL for 24 h, then cells were stimulated with IFN-*β* or SeV for 12 h, and their luciferase activities were detected. (E) The RNA levels of Cig5 and IFIT1 were detected in TRIM69 transfected 293T cells stimulated with SeV. NS, not significant. The data shown are representative of at least 3 independent experiments.
(TIF)

S6 Fig MS analysis of target proteins by TRIM69 co-IP assay in 293T infected with DENV-2. (A) The map showed distribution of IP proteins from Flag or TRIM69-Flag. (B) Target proteins immunoprecipitated by TRIM69-Flag were shown.
(TIF)

S7 Fig TRIM69 reduces the amount of NS2B3 and influences its function. (A) TRIM69 reduced the protein level of NS2B3 complex, thereby reduced the cleavage efficacy on STING. (B) Overexpression of TRIM69 did not interfere with the interaction between NS2B and NS3. Cells were co-transfected with NS2B, NS3 and TRIM69 (or control vector) for 48h, and then treated with MG132. The interaction between NS2B and NS3 were analyzed by immunoprecipitation and western blots.
(TIF)

S8 Fig mTRIM69 interacts with DENV NS3 in mouse cells. (A) Co-localization of mTRIM69-Myc (Green) and NS3-Flag (Red) in mouse B16F10 cells as analyzed by confocal microscopy. (B) Co-IP of endogenous mTRIM69 and NS3 from lysates of B16F10 cells infected with DENV-2 for 48 h.
(TIF)

S1 Table Induction of selected of well-known (A) and predicted ISGs (B) by DENV-2 infection. (DOCX)
