# Supplementary figures and images for: Interferon-stimulated TRIM69 interrupts dengue virus replication by ubiquitinating viral nonstructural protein 3
Source: PLoS Pathog. 2018 Aug 24;14(8):e1007287. doi: 10.1371/journal.ppat.1007287 (PMC6126873; doi:10.1371/journal.ppat.1007287)

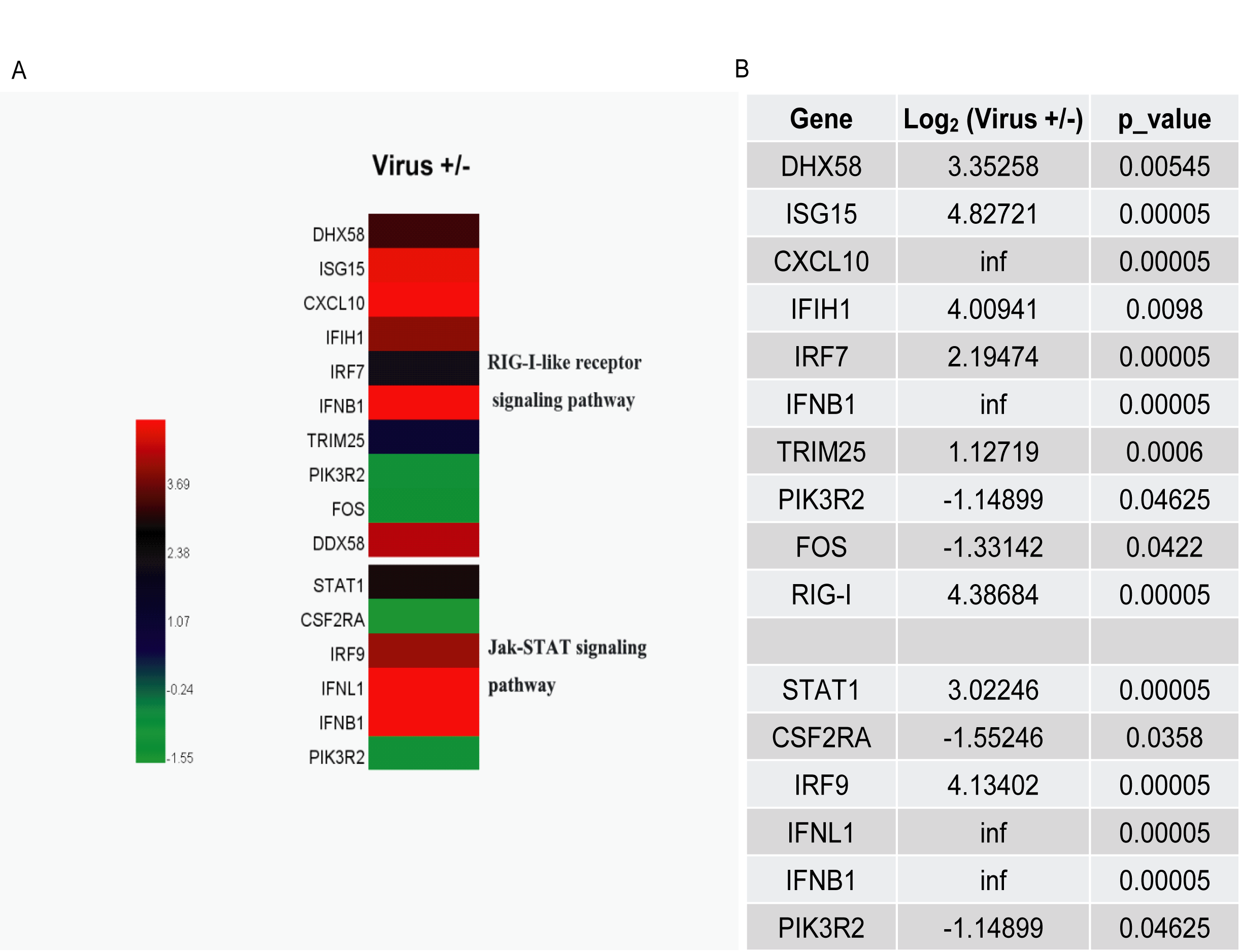

Supplement: S1 Fig — (A) Color intensity refers to the mean relative expression fold changes in comparison to non-infected cells. Red for upregulated, green for down regulated, and black for no change. (B) Fold change and p value of selected genes listed in panel A. (inf: infinity) (TIF) [file ppat.1007287.s001.tif]

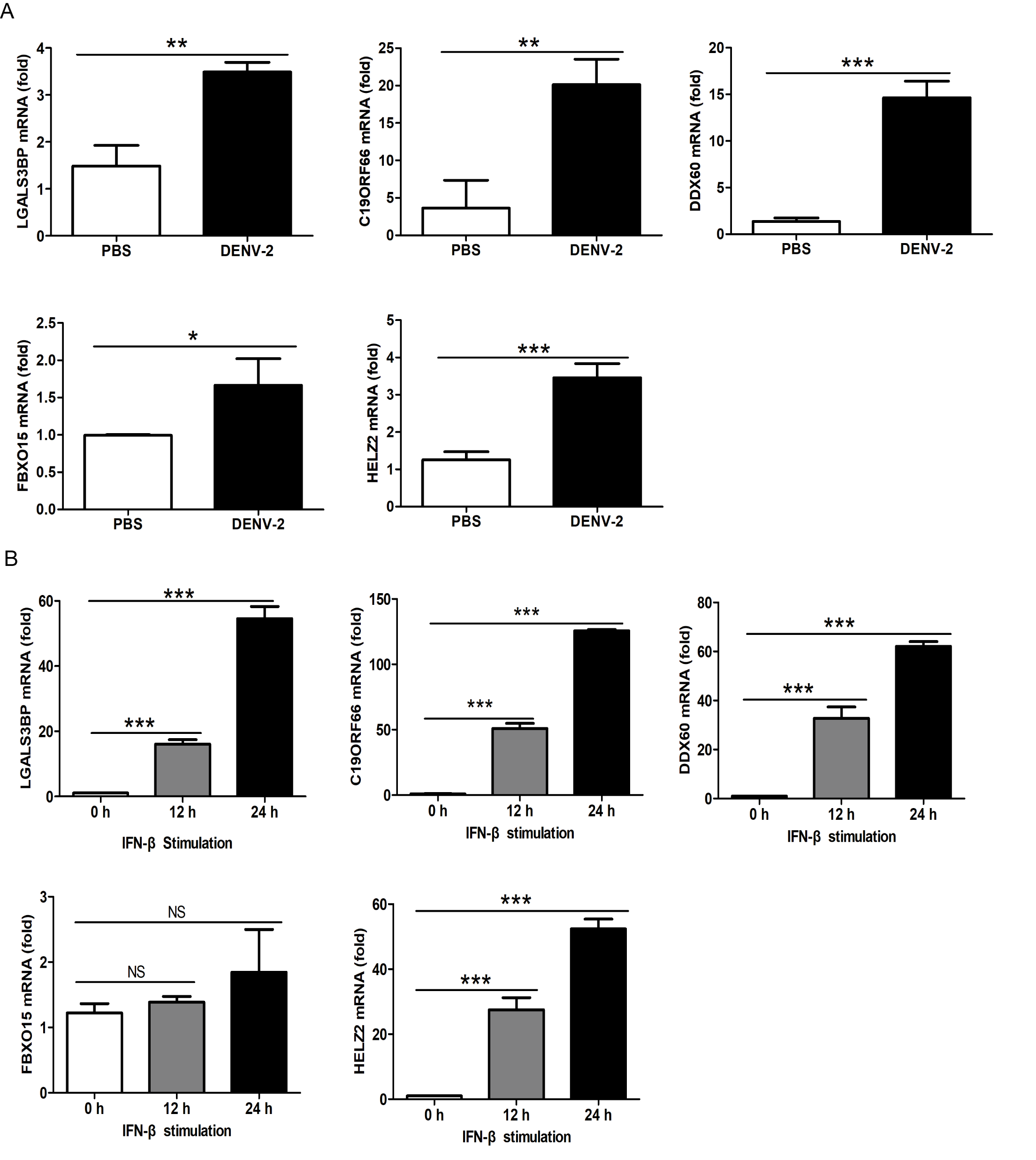

Supplement: S2 Fig — qTR-PCR analysis of 5 selected genes stimulated with DENV-2 (A) or IFN-β (B) in 293T cells. Results are expressed as mean ± SEM. NS, not significant.* p < 0.05, ** p < 0.01, and *** p < 0.001. The data shown are representative of at least 3 independent experiments. (TIF) [file ppat.1007287.s002.tif]

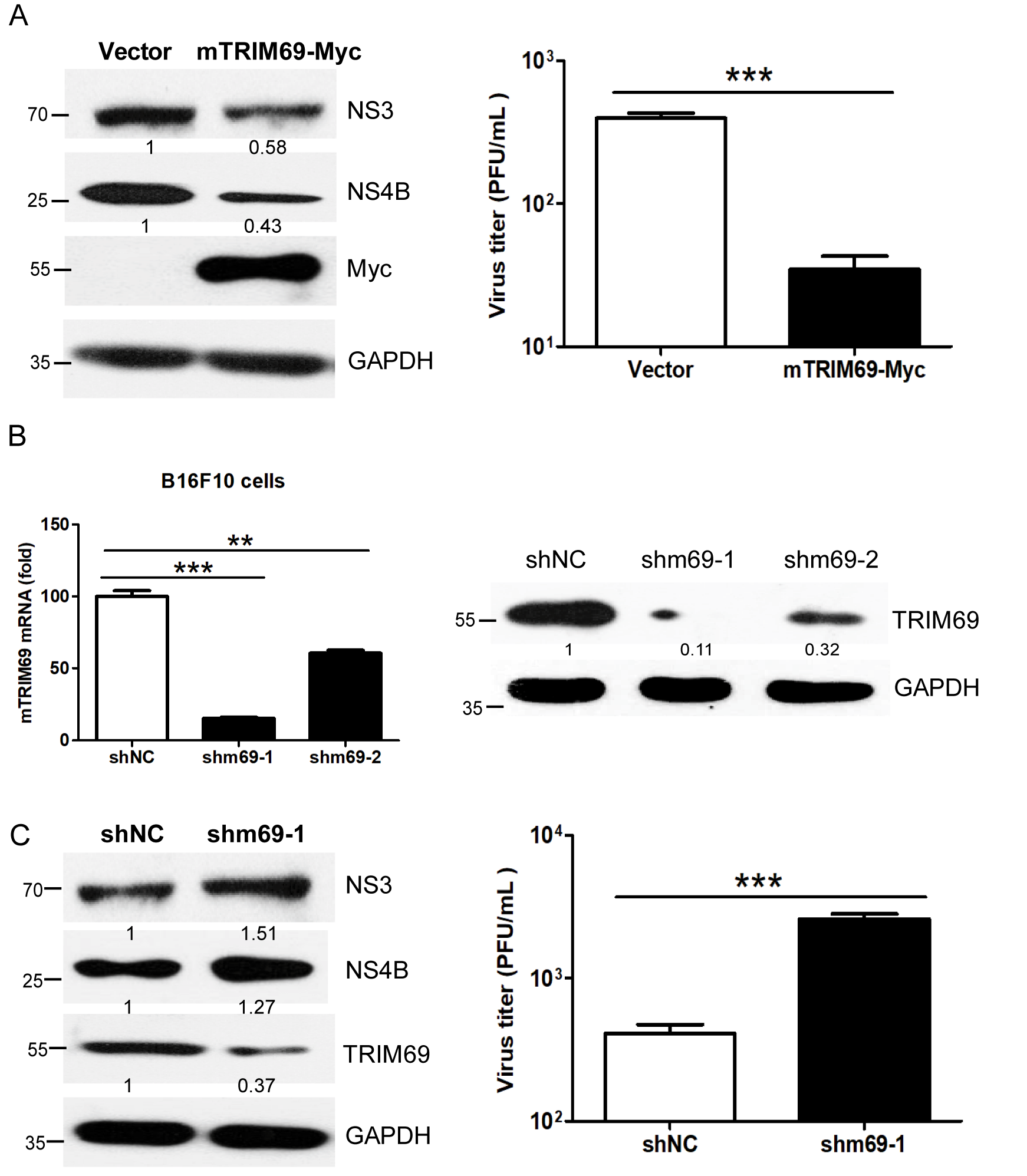

Supplement: S3 Fig — (A) mTRIM69-Myc was transfected into mouse B16F10 cells for 24 h. Then DENV-2 was infected the cells for another 24 h. Cell lysates and supernatants were harvested for Western blot and TCID50 assays, respectively. (B) The knockdown efficiency of two shRNAs (shm69-1 and shm69-2) targeting mouse TRIM69 was detected in B16F10 cells. The mRNA level (left) and protein (right) of mouse TRIM69 were analyzed. (C) The viral proteins and virus titers were tested in mTRIM69 silenced B16F10 cells. (TIF) [file ppat.1007287.s003.tif]

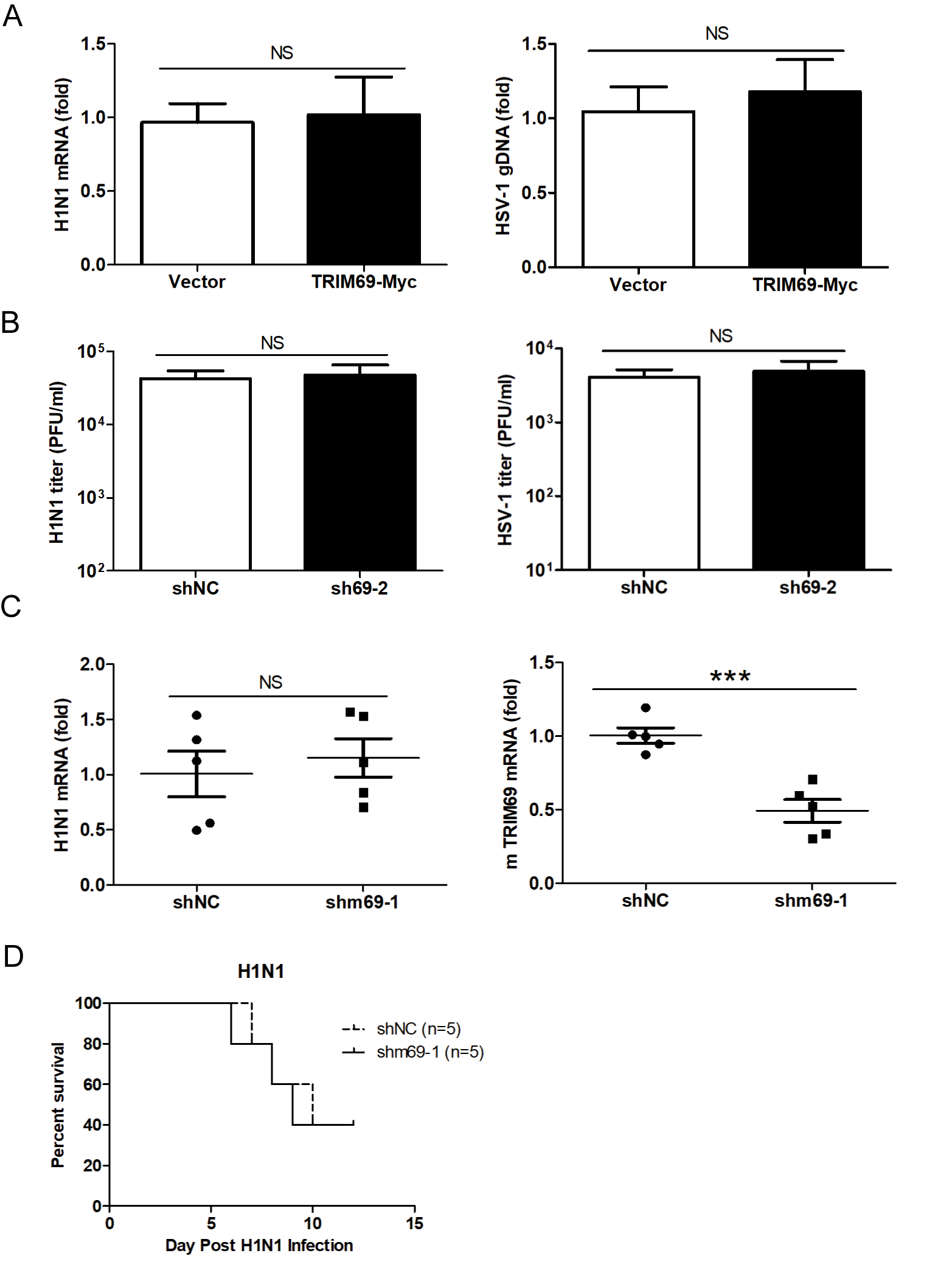

Supplement: S4 Fig — (A) H1N1 and HSV-1 nucleotide copies were comparable in TRIM69 overexpressed cells and control cells. (B) Viral titers of H1N1 and HSV-1 from supernatants of control or TRIM69 overexpressed cells. (C) Viral load of H1N1 in peripheral blood cells in control and TRIM69 silenced mice as determined by qRT-PCR (left). TRIM69 knockdown efficiency in peripheral blood cells were confirmed by qRT-PCR (right). NS, not significant. The data shown are representative of 3 independent experiments. (D) Survival curve of H1N1 infected wide type and TRIM69 silenced mice (n = 5). Mice were infected with intranasal infection of 2x10^5^ pfu H1N1and monitored daily for survival rates. (TIF) [file ppat.1007287.s004.tif]

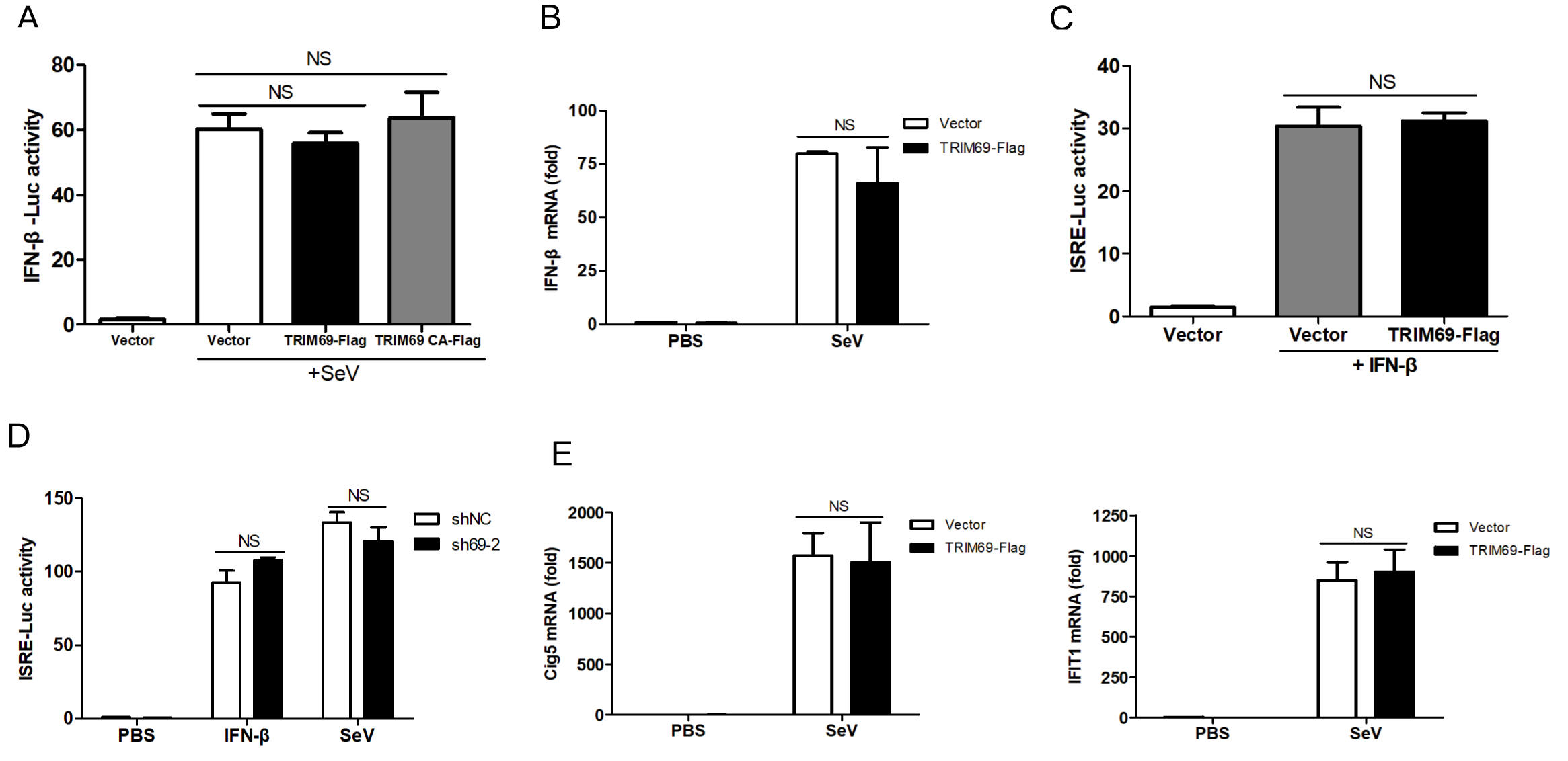

Supplement: S5 Fig — (A) TRIM69 or TRIM69 CA did not affect SeV-stimulated IFN-β activation. IFN-β-driven luciferase activity was determined by a dual-luciferase assay. (B) The RNA level of IFN-β was detected in TRIM69 transfected 293T cells stimulated with SeV. (C) TRIM69 overexpression did not influence IFN-β-stimulated ISRE promoter activation. 293T were treated with IFN-β for 12 h and harvested to test the ISRE-luciferase activity. (D) Knockdown of TRIM69 did not influence IFN-β or SeV-stimulated ISRE promoter activity. shNC or sh69-2 was co-transfected with ISRE-luc and pRL for 24 h, then cells were stimulated with IFN-β or SeV for 12 h, and their luciferase activities were detected. (E) The RNA levels of Cig5 and IFIT1 were detected in TRIM69 transfected 293T cells stimulated with SeV. NS, not significant. The data shown are representative of at least 3 independent experiments. (TIF) [file ppat.1007287.s005.tif]

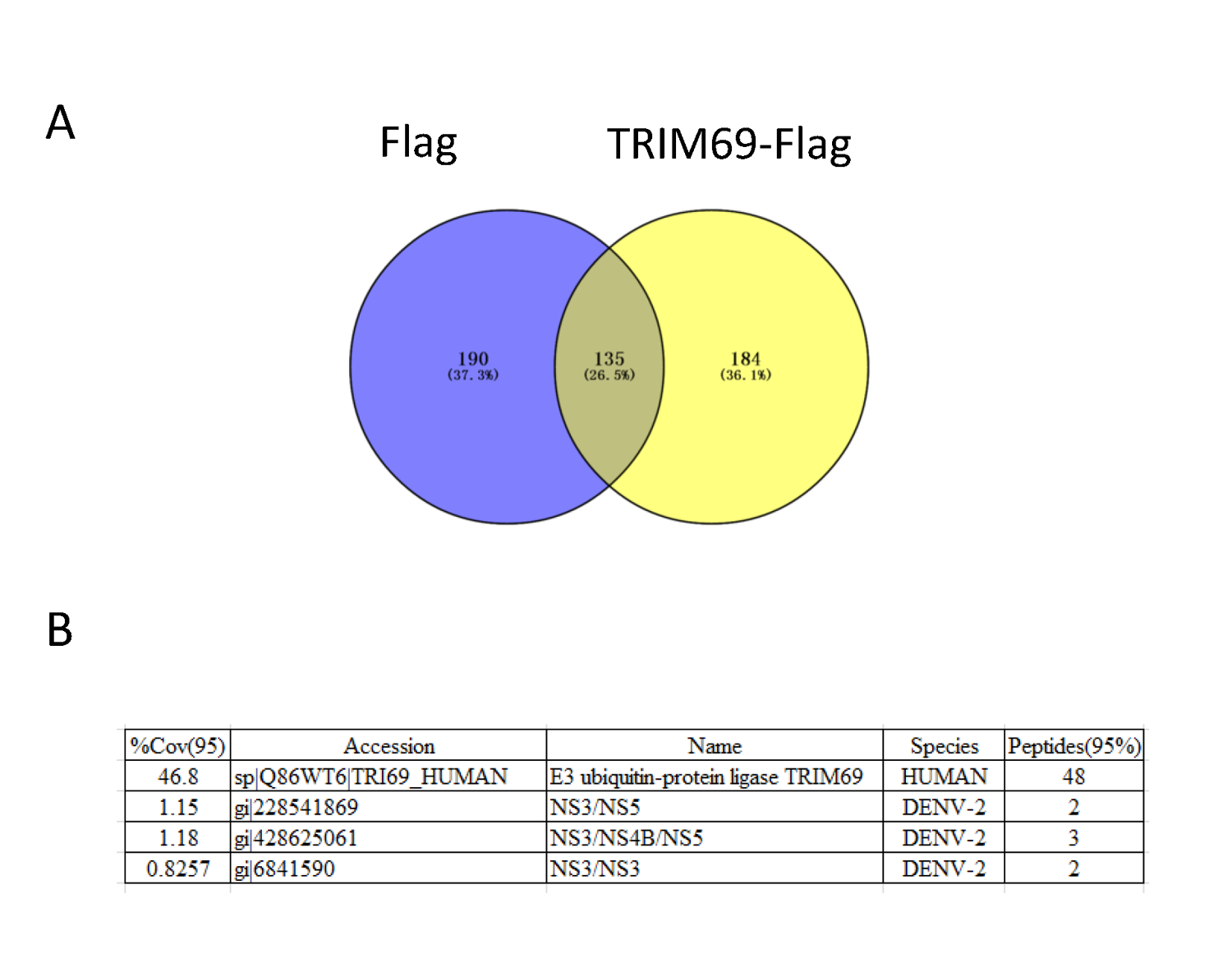

Supplement: S6 Fig — (A) The map showed distribution of IP proteins from Flag or TRIM69-Flag. (B) Target proteins immunoprecipitated by TRIM69-Flag were shown. (TIF) [file ppat.1007287.s006.tif]

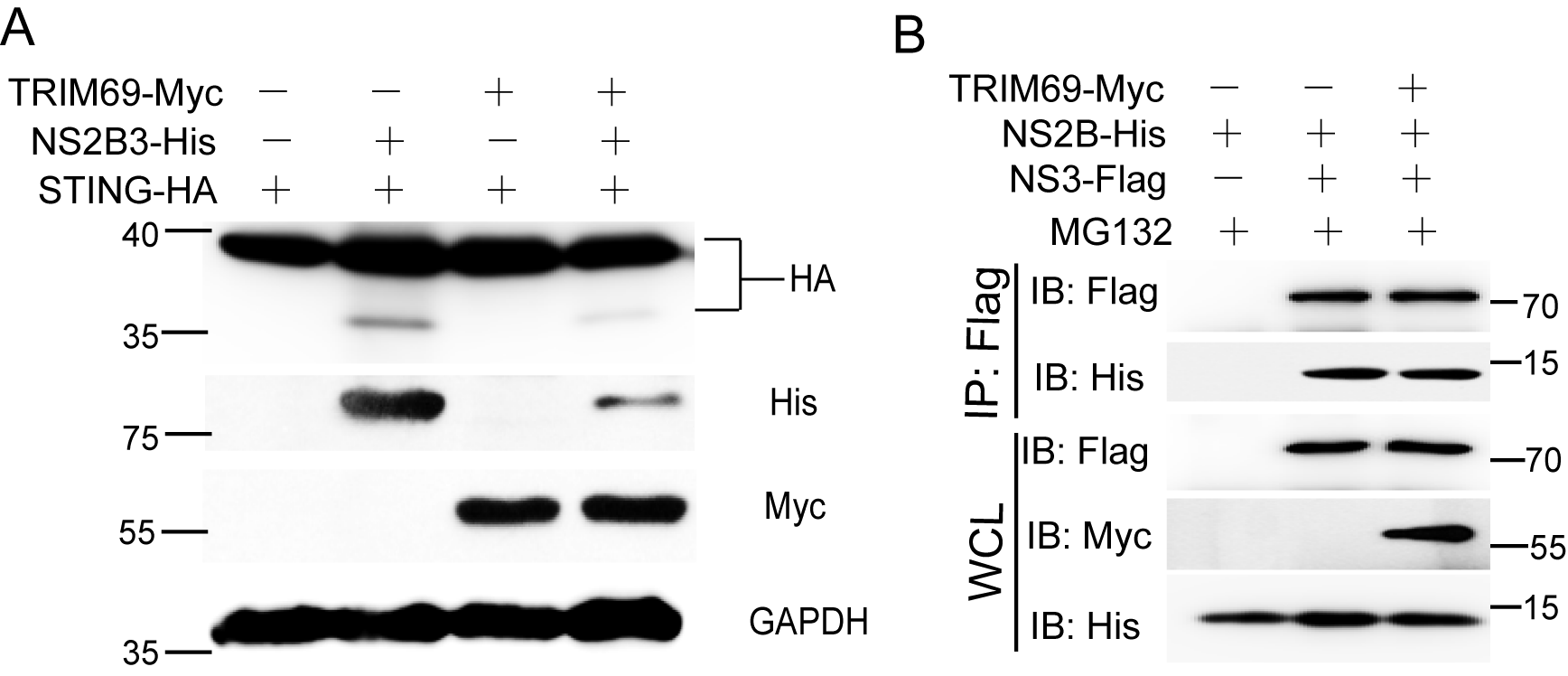

Supplement: S7 Fig — (A) TRIM69 reduced the protein level of NS2B3 complex, thereby reduced the cleavage efficacy on STING. (B) Overexpression of TRIM69 did not interfere with the interaction between NS2B and NS3. Cells were co-transfected with NS2B, NS3 and TRIM69 (or control vector) for 48h, and then treated with MG132. The interaction between NS2B and NS3 were analyzed by immunoprecipitation and western blots. (TIF) [file ppat.1007287.s007.tif]

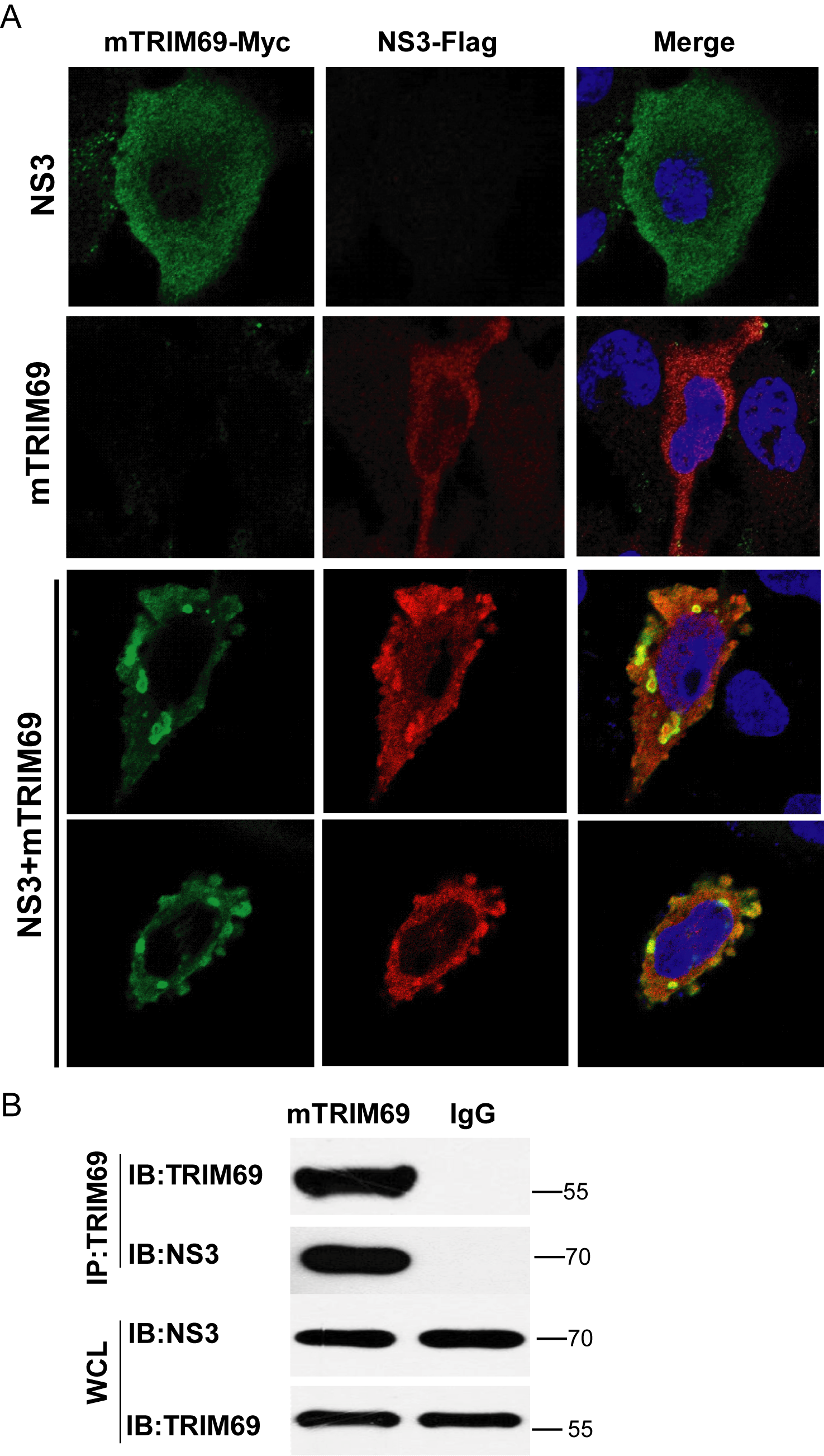

Supplement: S8 Fig — (A) Co-localization of mTRIM69-Myc (Green) and NS3-Flag (Red) in mouse B16F10 cells as analyzed by confocal microscopy. (B) Co-IP of endogenous mTRIM69 and NS3 from lysates of B16F10 cells infected with DENV-2 for 48 h. (TIF) [file ppat.1007287.s008.tif]

A


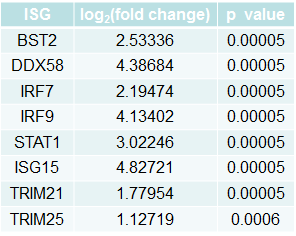


B


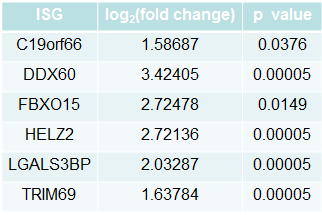

Supplement: S1 Table — (DOCX) [file ppat.1007287.s009.docx]
